# Multi-omics single-cell data alignment and integration with enhanced contrastive learning and differential attention mechanism

**DOI:** 10.1093/bioinformatics/btaf443

**Published:** 2025-08-07

**Authors:** Tianjiao Zhang, Zhongqian Zhao, Hongfei Zhang, Zhenao Wu, Fang Wang, Guohua Wang

**Affiliations:** College of Computer and Control Engineering, Northeast Forestry University, Harbin, 150040, China; College of Computer and Control Engineering, Northeast Forestry University, Harbin, 150040, China; College of Computer and Control Engineering, Northeast Forestry University, Harbin, 150040, China; College of Computer and Control Engineering, Northeast Forestry University, Harbin, 150040, China; The Quzhou Affiliated Hospital of Wenzhou Medical University, Quzhou People's Hospital, Quzhou, 324000, China; College of Computer and Control Engineering, Northeast Forestry University, Harbin, 150040, China; Faculty of Computing, Harbin Institute of Technology, Harbin, 150001, China

## Abstract

**Motivation:**

Identifying cell types that constitute complex tissue components using single-cell sequencing data is a critical issue in the field of biology. With the continuous advancement of sequencing technologies, the recognition of cell types has evolved from analyzing single-omics scRNA-seq data to integrating multi-omics single-cell data. However, existing methods for integrative analysis of high-dimensional multi-omics single-cell sequencing data have several limitations, including reliance on specific distribution assumptions of the data, sensitivity to noise, and clustering accuracy constrained by independent clustering methods. These issues have restricted improvements in the accuracy of cell type identification and hindered the application of such methods to large-scale datasets for cell type recognition. To address these challenges, we propose a novel method for aligning and integrating single-cell multi-omics data—scECDA.

**Results:**

The scECDA employs independently designed autoencoders that can autonomously learn the feature distributions of each omics dataset. By incorporating enhanced contrastive learning and differential attention mechanisms, the scECDA effectively reduces the interference of noise during data integration. The model design exhibits high flexibility, enabling adaptation to single-cell omics data generated by different technological platforms. It directly outputs integrated latent features and end-to-end cell clustering results. Through the analysis of the distribution of latent features, the scECDA can effectively identify key biological markers and precisely distinguish cell subtypes, recover cluster-specific motif and infer trajectory. The scECDA was applied to eight paired single-cell multi-omics datasets, covering data generated by 10X Multiome, CITE-seq, and TEA-seq technologies. Compared to eight state-of-the-art methods, scECDA demonstrated higher accuracy in cell clustering.

**Availability and implementation:**

The scECDA code is freely available at https://github.com/SuperheroBetter/scECDA

## 1 Introduction

Single-cell multi-omics sequencing technologies have emerged as powerful tools for capturing the complex heterogeneity of cells. These technologies enable the simultaneous measurement of gene expression, chromatin accessibility, and protein abundance at single-cell resolution, thereby facilitating a more comprehensive analysis of cellular states and regulatory mechanisms. For instance, single-cell RNA sequencing (scRNA-seq) has been widely applied across diverse tissues and disease contexts, where the investigation of gene expression profiles allows for the identification of marker genes and the exploration of intercellular and intergenic relationships. Single-cell assay for transposase-accessible chromatin sequencing (scATAC-seq) leverages the transcriptional activity state of chromatin to identify regulatory elements and infer cellular differentiation trajectories. Antibody-based methods, such as Cellular Indexing of Transcriptomes and Epitopes by Sequencing (CITE-seq) and 10X Multiome, further expand the scope of these technologies by integrating protein abundance data, enabling in-depth studies of cell surface markers, signaling pathways, and immune cell phenotypes. The integration of multi-omics data serves as the initial stage of joint analysis, aiming to align data from different omics layers into a unified latent feature space. Such integration can provide deeper insights into cell-specific regulatory networks by inferring upstream regulatory factors (Miao *et al.* 2021) and contribute to the identification of additional cell clusters and biomarkers (Jin *et al.* 2020).

Researchers have developed numerous computational tools for integrating single-cell multi-omics data, which can be broadly categorized into three classes: Anchor-based alignment methods: These approaches leverage mutual nearest neighbors (MNN) or statistical techniques to identify cross-modal anchors for data integration. For example, Seurat v3 (Stuart *et al.* 2019) employs canonical correlation analysis (CCA) combined with MNN to detect anchors, while MOJITOO (Cheng *et al.* 2022) effectively infers shared representations across multiple modalities using CCA. Matrix factorization-based methods: These techniques extract common patterns from different omics layers via matrix or tensor factorization. iNMF (Lee and Seung 1999) extends non-negative matrix factorization (NMF) to multi-omics data, enabling more precise identification of cell clusters. Mowgli (Huizing *et al.* 2023) integrates iNMF with optimal transport to capture inter-omics relationships and improve fusion quality. Deep learning models based on (variational) autoencoders: These frameworks employ encoder architectures to map heterogeneous omics data into a unified latent space. For instance, scMVP (Li *et al.* 2022) introduces a clustering-consistent constrained multi-view variational autoencoder (VAE) to learn a shared latent representation while using separate decoders to reconstruct each omics layer. Omics-specific distributional assumptions: TotalVI (Gayoso *et al.* 2021) models RNA-seq data using a negative binomial distribution and antibody-derived tag (ADT) data via a negative binomial mixture model, subsequently learning a cross-omics low-dimensional representation. Despite their contributions, these methods exhibit notable limitations: Low-quality data may lead to erroneous anchor alignment in anchor-based methods or introduce noise in the unified latent space derived from NMF, compromising integration accuracy. Most models (e.g. scMVP) lack scalability for integrating three or more omics layers. Evaluation of multi-omics integration models often relies on complex downstream workflows. For example, after obtaining latent representations via Mowgli, clustering algorithms such as Leiden (Traag *et al.* 2019) or K-means (Hartigan and Wong 1979) are applied. However, this introduces critical challenges: The involvement of multiple clustering methods increases analytical complexity. Clustering performance varies with parameter selection, data characteristics, and algorithmic biases, complicating result interpretation. Crucially, this pipeline obscures performance attribution—whether superior outcomes stem from the integration model (e.g. Mowgli) or the clustering algorithm—thereby impeding objective model assessment. These technical constraints not only hinder interpretability but may also lead to misjudgements of a model’s integration capability.

Given the limitations of existing methods and the inherent high-dimensionality and sparsity of single-cell omics data, this study proposes scECDA, a novel approach for single-cell multi-omics data alignment and integration. To mitigate the impact of noisy data on clustering results, scECDA incorporates a differential attention mechanism (Ye *et al.* 2025) and introduces a feature fusion module that automatically enhances the signal-to-noise ratio of biologically relevant features. Furthermore, to align different omics profiles from the same cell into a unified feature space, scECDA employs contrastive learning alongside a simple yet effective data augmentation strategy to generate positive and negative training samples. During inference, the model directly outputs both the integrated latent representation of multi-omics data and the final cell clustering assignments. To evaluate scECDA’s performance, we benchmarked it against eight state-of-the-art single-cell omics integration methods—TriTan (Ma *et al.* 2025), Mowgli Huizing *et al.* 2023), MOJITOO, scMVP, scDMSC (Wang *et al.* 2025), scMCs (Ren *et al.* 2023), K-means (Hartigan and Wong 1979), scRISE(Xie *et al.* 2024) — across eight datasets with diverse characteristics. Among these: TriTan and Mowgli, based on non-negative matrix factorization (NMF), effectively integrate three or more omics layers. MOJITOO seeks the optimal subspace based on CCA. These three methods can effectively integrate three or more omics data. scMVP (Multi-view variational autoencoder model with clustering consistency constraints), scDMSC (multi-view subspace learning) and scMCs (optimized subspace clustering) specialize in dual-omics integration. scRISE (based on a graph autoencoder) and K-means support only single-omics clustering. These methods are introduced in detail in the Supplementary Materials (P22), available as supplementary data at *Bioinformatics* online. Experimental results demonstrate that scECDA consistently outperforms competing methods across datasets of varying types and dimensionalities, showcasing its robustness and versatility in single-cell multi-omics integration.

## 2 Materials and methods

**Figure 1.**
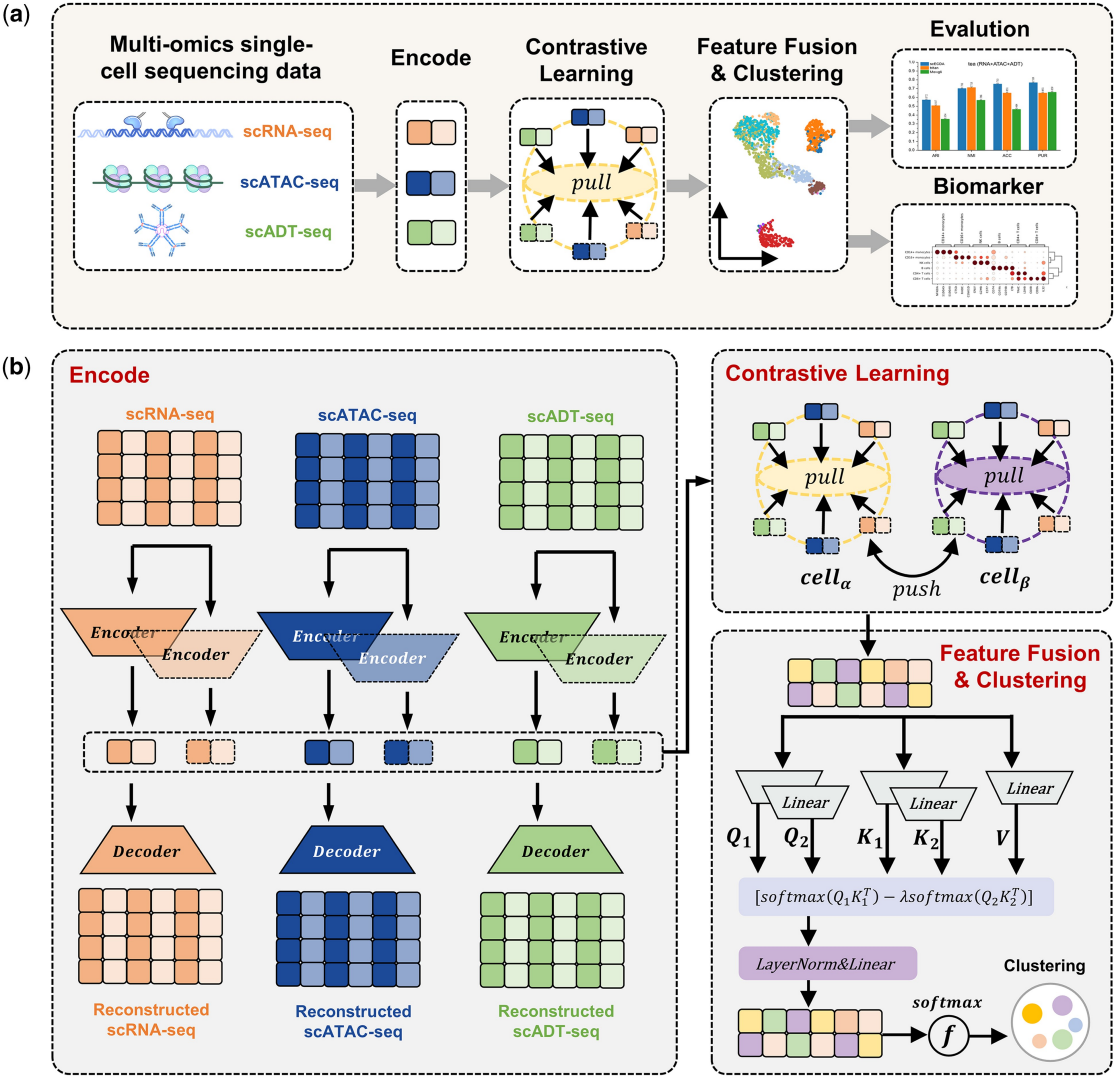
scECDA method. (a) Illustrates the compositional structure of the input module, which receives paired single-cell multimodal omics data (including scRNA-seq, scATAC-seq, and scADT-seq. These figures were made from https://www.cnsknowall.com). It performs multimodal data integration and joint clustering through deep neural networks. As shown in (b), the framework’s specific implementation includes the following key steps: First, individual encoder architectures are used to extract low-dimensional latent representations from each single-cell omics data type, which are iteratively optimized using reconstruction loss functions. Next, an auxiliary encoder is constructed using a parameter-sharing mechanism, where controlled perturbations are applied to the latent space features to generate augmented samples. During the feature alignment phase, a contrastive learning loss function is used to constrain the latent representations of different omics data from the same cell, maximizing their cosine similarity in the shared embedding space. After alignment, the cross-omics features are concatenated and input into a differential attention module, which enhances the signal-to-noise ratio of effective features. Finally, the fused features are passed through a clustering module to produce the cell clustering results.

The scECDA method employs a modularized computational pipeline (as shown in Fig. 1), primarily consisting of three core stages: multi-omics feature extraction, latent feature alignment and fusion, and clustering analysis. Initially, the method constructs independent deep encoder frameworks to extract low-dimensional latent representations specific to each omics dataset from the raw single-cell multi-omics data, thereby preserving the uniqueness of each dataset. Subsequently, to mitigate noise interference and enhance the consistency of feature representations, smoothing processing is applied to the extracted latent representations. During the latent feature alignment stage, scECDA incorporates a contrastive learning strategy (contrastive learning), optimizing the distance metric between positive and negative sample pairs to achieve semantic alignment of cross-omics latent representations. To further enhance the integration of multi-omics data, the method introduces a differential attention mechanism and designs a feature fusion module. This module adaptively weights the contributions of features from each omics dataset, generating unified latent representations with improved discriminability. Finally, unsupervised clustering analysis is performed on the fused latent representations to comprehensively integrate and clustering single-cell multi-omics data.

### 2.1 Encode single-cell omic data

Dataset definition. Let the dataset be defined as $X^{u}\in \{X^{1},X^{2},\ldots ,X^{v}\}$, where $X^{u}\in R^{n\times m^{u}}$, representing n cells and $m^{u}$ features for each omics dataset u. Each dataset $X^{u}$ consists of n samples, denoted as $X^{u}=\{x_{1}^{u},x_{2}^{u},\ldots ,x_{n}^{u}\}$, where i∈[1, n] and u∈[1, v].

Model design. Given that the feature distribution of each omics dataset differs, this study designs an autoencoder for each dataset to perform dimensionality reduction and denoising. The goal is to preserve the most critical features of each dataset. The latent features extracted by the encoder can be represented as:


(1) Zu=Eu(Xu;ΘEu)

where $E^{u}$ represents the encoder for the u-th omics dataset, with parameters $\Theta_{E}^{u}$. The input dimension $m^{u}$ is reduced to $d^{u}$, resulting in $Z^{u}\in R^{n\times d^{u}}$, denoted as $Z^{u}=\{z_{1}^{u},z_{2}^{u},\ldots ,z_{n}^{u}\}$. The decoder reconstructs the data ${\hat{X}}^{u}=D^{u}(Z^{u};\Theta_{D}^{u})$, $\Theta_{D}^{u}$ represents the parameters of the decoder. The autoencoder is trained by minimizing the mean squared error (MSE) between the input and reconstructed data:


(2) Lreconstructionu=∥Xu-X^u∥2

(3) Lstage1=∑k=1vLreconstructionk

where $∥\cdot {∥}_{2}$ denotes the $\ell_{2}$ norm.

### 2.2 Denoising of latent features

In single-cell multi-omics data analysis, scATAC-seq data, characterized by its high dimensionality and sparsity, introduces noise during the extraction of latent features. If this noise is not properly addressed, it can directly degrade the quality of the latent features, leading to systematic biases in subsequent clustering analyses. To mitigate this challenge, there is an urgent need to develop a feature distribution estimation method that possesses smoothness and robustness, thereby reducing the adverse effects of low-quality data on clustering results. In response to this challenge, this study employs a Student’s t-distribution to perform spatial transformation on the latent features $Z^{u}$:


(4) qiju=(1.0+∥ziu-ξju∥)-1∑j=1k (1.0+∥ziu-ξju∥)-1

Specifically, this study first applies the K-means algorithm to the latent features $\left\{z_{1}^{u},z_{2}^{u},\ldots ,z_{n}^{u}\right\}$ of the u-th omics data to perform clustering analysis and obtain the corresponding clustering centers $\xi_{j}^{u}$, which serve as reference points for feature transformation. This transformation strategy based on the t-distribution enhances the stability of feature representation, thereby improving the accuracy and reliability of subsequent analyses.

To obtain more accurate clustering centers $\xi_{j}^{u}$, this study proposes a robust method for evaluating the quality of latent features based on principal component analysis (PCA) and the interquartile range (IQR) criterion, aimed at filtering high-quality single-cell data. Specifically, the latent feature matrix $z_{i}^{u}$ is first dimensionally reduced by projecting it into a 40-dimensional principal component space: $h_{i}^{u}=\text{PCA}(z_{i}^{u})\in R^{n\times 40}$. Subsequently, the Euclidean distance matrix $D\in R^{n\times n}$ is computed in the low-dimensional space, where $D_{\mathrm{ij}}$ represents the distance between the i-th and j-th cells. The minimum distance for each cell to other cells is preserved as $\mathrm{mdis}=\underset{i}{\min}\;D_{\cdot j}\in R^{n}$.

To identify outlier cells, the IQR criterion is used to define a threshold: $\text{threshold}=Q_{3}+1.5\times \text{IQR}$, where $Q_{3}$ is the third quartile (75th percentile) of the nearest neighbor distances, $\text{IQR}=Q_{3}-Q_{1}$ is the interquartile range, and $Q_{1}$ is the first quartile (25th percentile). Finally, cells with mdis values greater than the threshold are classified as outliers, while the remaining cells are retained as high-quality cells. Through this filtering process, most noise in the latent feature space can be removed, providing a more reliable subset of cells for downstream analyses. Further explanations can be found in the Supplementary Materials (P25), available as supplementary data at *Bioinformatics* online.

### 2.3 Alignment of latent features across omics data

This study employs a contrastive learning framework (Chen *et al.* 2020) to align latent features $q_{\mathrm{ij}}^{u}$ across different omics data. Inspired by Monae (Tang *et al.* 2024), we design a data augmentation module to more effectively learn robust feature representations during the contrastive learning process, thereby improving model performance on downstream tasks. Data augmentation is one of the key strategies to enhance model performance. Traditional augmentation methods, such as flipping images, cropping, or word masking in text, expand sample size by introducing perturbations in the original data space. However, these methods have significant limitations: first, they heavily rely on domain-specific prior knowledge; second, they are difficult to apply across domains, limiting their generalizability. Compared to traditional methods that operate directly in the original data space, researchers have recently proposed more generalizable strategies for perturbing latent space features. These methods have three notable advantages: (i) they transcend domain limitations and can be widely applied to any type of dataset; (ii) they are straightforward to implement without requiring complex preprocessing steps; and (iii) they do not introduce additional neural network parameters. Recent studies (Gao *et al.* 2021) have demonstrated the excellence of latent space feature perturbation in improving model generalization.

In single-cell multi-omics data analysis, traditional data augmentation methods (e.g. randomly zeroing out elements of the data matrix) may disrupt the expression patterns of key genes or peak features in cells, leading to the loss of important biological information. Specifically, single-cell RNA and ATAC data are characterized by high sparsity and high dimensionality, where non-zero values may carry critical biological meanings, such as specific gene expression levels or signal intensities of chromatin accessibility regions. If a simple random zeroing strategy is applied, it may negatively impact the following aspects: (i) the expression levels of key regulatory genes may be erroneously suppressed, affecting downstream cell type identification; (ii) important signals from open chromatin regions may be weakened, interfering with the recognition of cis-regulatory elements; and (iii) the heterogeneity features between cells may be disrupted, reducing the accuracy of cell state clustering. Therefore, this study adopts a latent feature perturbation approach for data augmentation.

We construct an auxiliary encoder ${\tilde{E}}^{u}$ identical in structure to the original encoder $E^{u}$, where both share the same neural network architecture and parameters (as shown in Fig. 1). To achieve feature perturbation, ${\tilde{E}}^{u}$ adds a Dropout layer on top of the original structure and injects a small perturbation ε to simulate varying sequencing depths in real-world scenarios. This design ensures compatibility between the feature spaces of the encoders while generating diversified feature representations through controlled perturbations, thereby enhancing the model’s generalization ability. The specific process is as follows: ${\tilde{Z}}^{u}={\tilde{E}}^{u}(X^{u};\Theta_{E}^{u})+\varepsilon =\{{\tilde{z}}_{1}^{u},{\tilde{z}}_{2}^{u},\ldots ,{\tilde{z}}_{n}^{u}\}$, ${\tilde{q}}_{\mathrm{ij}}^{u}=\frac{(1.0+∥{\tilde{z}}_{i}^{u}-\xi_{j}^{u}∥{)}^{-1}}{\sum_{j=1}^{k}\;(1.0+∥{\tilde{z}}_{i}^{u}-\xi_{j}^{u}∥{)}^{-1}}$. The set of positive sample pairs for cell i is constructed as $S_{i}^{+}=\overset{v}{\underset{u=1}{\cup }}\overset{v}{\underset{l=u+1}{\cup }}〈q_{i\cdot }^{u},q_{i\cdot }^{v}〉\cup \overset{v}{\underset{u=1,\;l=1,\;u\neq l}{\cup }}\left\langle {\tilde{q}}_{i\cdot }^{u},q_{i\cdot }^{v}\right\rangle$, where ⟨⋅,⋅⟩ denotes a sample pair, and ∪ represents the union operation. Within the same batch, the latent features of other cells k and their related positive samples are considered as negative samples for cell i( i≠k), with b cells per batch: $S_{i}^{-}=\overset{v}{\underset{u=1}{\cup }}\overset{v}{\underset{l=1}{\cup }}\overset{b}{\underset{k=1,k\neq i}{\cup }}\left(\left\langle q_{i\cdot }^{u},q_{k\cdot }^{l}\rangle \cup \langle {\tilde{q}}_{i\cdot }^{u},q_{k\cdot }^{l}\rangle \cup \langle q_{i\cdot }^{u},{\tilde{q}}_{k\cdot }^{l}\right\rangle \right)$. The distance $d_{\left(\cdot ,\cdot \right)}^{+}$ between cell i and its positive samples in the latent space is calculated as $d_{i,i^{+}}^{+}=\frac{{{(\beta }_{i})}^{T}\cdot \beta_{i^{+}}\;}{\tau \|\beta_{i}\|\|\beta_{i^{+}}\|}$, where $i^{+}$ is the positive sample of cell i, $\langle \beta_{i},\beta_{i^{+}}\rangle \in S_{i}^{+}$, and τ is a tuning coefficient with a default value of 1.0. The distance $d_{\left(\cdot ,\cdot \right)}^{-}$ between cell i and all negative samples in the latent space is calculated as $d_{i,i^{-}}^{-}=\sum_{i^{-}}\frac{{{(\beta }_{i})}^{T}\cdot \beta_{i^{-}}\;}{\tau \|\beta_{i}\|\|\beta_{i^{-}}\|}$, where $i^{-}$ is the negative sample of cell i, and $\langle \beta_{i},\beta_{i^{-}}\rangle \in S_{i}^{-}$. Thus, the contrastive loss can be expressed as $L_{\mathrm{entropy}}=\sum_{u=1}^{v}\sum_{j=1}^{b}\rho_{j}^{u}l\mathrm{og}\rho_{j}^{u}$, where $\rho_{j}^{u}=\frac{1}{b}\sum_{i=1}^{b}q_{\mathrm{ij}}^{u}$. The regularized contrastive loss function is


(5) Lstage2=Lcontrastive+Lentropy

### 2.4 Integration of omics data features

In single-cell omics studies, the information provided by single-omics data is limited. In comparison, the integration of multi-omics data enables complementary information, providing a more comprehensive perspective for downstream analysis tasks such as biomarker identification and cell clustering. In the aforementioned steps, we have smoothed and aligned the latent features of each omics data to minimize the interference of noise on subsequent calculations. To further enhance the integration effect of multi-omics data, this study introduces the differential attention mechanism (Ye *et al.* 2025) for the first time and designs a module for fusing single-cell multi-omics data features. Compared to the traditional self-attention mechanism (Vaswani *et al.* 2017), the differential attention mechanism strengthens the expression weights of key feature information and weakens the influence of irrelevant feature information, thereby more effectively capturing the global structural relationships of omics data during the fusion of single-cell multi-omics data. It significantly increases the proportion of key feature information. In contrast, the self-attention mechanism has the limitation of over-allocating attention scores to irrelevant feature information, potentially causing key information to be lost or weakened during feature fusion. Therefore, this study combines the characteristics of the differential attention mechanism with the specific requirements of integrating single-cell multi-omics data, providing a more effective solution. The specific process is as follows: First, concatenate the latent features of each omics data obtained from Equation (1): $Z=[Z^{1},Z^{2},\ldots ,Z^{u}]$, where $Z\in R^{n\times \sum_{u=1}^{v}d^{u}}$. Second, project Z into different feature spaces $[G_{1};G_{2}]=ZW_{1},\;[K_{1};K_{2}]=ZW_{2},\;Z^{\prime }=ZW_{3}$, where $W_{1},W_{2},W_{3}\in R^{\sum_{u=1}^{v}d^{u}\times 2f}$ are parameter matrices, with 2f=256 by default, and $G_{1},G_{2},K_{1},K_{2}\in R^{n\times f}$. Third, based on the differential attention computation formula: $O=\{\mathrm{softmax}(\frac{G_{1}K_{1}^{T}}{\sqrt{f}})-\lambda \mathrm{softmax}(\frac{G_{2}K_{2}^{T}}{\sqrt{f}})\}Z^{\prime }$, where λ is a learnable scalar: $\lambda =e^{\lambda_{g1}\cdot \lambda_{k1}}-e^{\lambda_{g2}\cdot \lambda_{k2}}+0.8-0.6\times e^{{-0.3\cdot \lambda }_{0}}$. where $\lambda_{g1},\lambda_{g2},\lambda_{k1},\lambda_{k2}\in R^{f}$ are learnable vectors, and $\lambda_{0}\in (0,10)$ is a constant used to initialize λ, with $\lambda_{0}=5$ in this study. Finally, after normalization, residual connection, and linear transformation, the final fused feature H is obtained: $O^{\prime }=\mathrm{Norm}(Z+O)$, $H=\mathrm{Norm}({O^{\prime }W}_{4}+O^{\prime })$, where $W_{4}\in R^{\sum_{u=1}^{v}d^{u}\times \sum_{u=1}^{v}d^{u}}$ is a parameter matrix for feature transformation of $O^{'}$.

### 2.5 Clustering module

The clustering module is responsible for dividing cell types based on the results of multi-omics feature fusion H. First, it calculates the probability of each cell belonging to each category: $A=\mathrm{softmax}(HW_{5})$, where $W_{5}\in R^{\sum_{u=1}^{v}d^{u}\times y}$ is a parameter matrix, and y represents the number of predefined categories. $C_{\mathrm{ij}}$ denotes soft clustering. To enhance the discriminability of the soft clustering results, the target distribution is constructed as follows:


(6) Pij=Aij2/∑i=1nAij∑j=1y(Aij2/∑i=1nAij)

where P is the clustering allocation probability matrix with enhanced category discriminability. The final category label for each cell is determined by taking the index of the maximum value in the probability matrix: $c_{i}=\underset{j}{\mathrm{argmax}}P_{i\cdot }$

### 2.6 Optimization objective

After obtaining the target distribution $P_{\mathrm{ij}}$ (Equation 6) and the distribution $q_{\mathrm{ij}}^{u}$ (Equation 4) of specific multi-omics data, the following loss function is used to guide $P_{\mathrm{ij}}$ with $q_{\mathrm{ij}}^{u}$:


(7) Lstage3=1v∑u=1vKL(P||qu)

where KL(⋅||⋅) represents the Kullback-Leibler (KL) divergence.scEDCA implements feature extraction and modality alignment through a multi-objective joint optimization framework. The first part ensures that the latent space effectively preserves the biological feature information of the original multi-omics data by minimizing the reconstruction loss $L_{\mathrm{stage}1}$ (Equation 3). The second part introduces the contrastive loss $L_{\mathrm{stage}2}$ (Equation 5) to align cross-omics data features by maximizing the similarity of feature representations from different omics data of the same cell. The third part uses KL divergence loss $L_{\mathrm{stage}3}$ (Equation 7) to constrain the distribution of the latent space, ensuring consistency between the target distribution and the prior distribution of specific multi-omics data. The total loss function L of the model is defined as a linear combination of these losses (as shown in Equation 26). The Adam algorithm is used for optimization, and the network parameters are updated iteratively through backpropagation. The model training process continues until the clustering accuracy converges, at which point the training is terminated. This multi-objective joint optimization achieves effective dimensionality reduction and denoising of multi-omics features, semantic alignment of cross-omics data features, and fusion of multi-omics feature distributions through probabilistic distribution guidance.


(8) L=αLstage1+βLstage2+γLstage3

When α=1, β=1, γ=0.5, the vast majority of datasets achieve good scores. The relevant experimental results are shown in Supplementary Fig. S20, available as supplementary data at *Bioinformatics* online.

### 2.7 Dataset collection and preprocessing

This study obtained nine real multi-omics datasets from the GEO database and previous research papers, with detailed information shown in Supplementary Table S1, available as supplementary data at *Bioinformatics* online. These datasets are categorized into three types based on the included omics types: paired scRNA-seq data with scATAC-seq data, paired scRNA-seq data with scADT-seq data, and paired scRNA-seq, scATAC-seq, and scADT-seq data.

In this study, we performed pre-processing on the single-cell multi-omics data. Specifically, for scRNA-seq and scATAC-seq data, we first filtered out genes expressed in fewer than two cells. Subsequently, we used scanpy (Wolf *et al.* 2018) toolkit to retain approximately 4000 highly variable genes in the RNA data and approximately 2000 highly variable genes in the ATAC data. To eliminate technical biases and enhance data comparability, we applied log normalization to both types of data, followed by data scaling. Furthermore, log normalization and standardization were also applied to scADT-seq data. These standardization steps help reduce technical variability across datasets, laying a foundation for subsequent multi-omics integration analysis.

### 2.8 Evaluation metrics

This study employs four widely-used evaluation metrics to assess performance: ARI, NMI, ACC, PUR, cASW, and cLISI.

The adjusted Rand index (ARI) is a measure of the consistency between clustering results and true cluster partitions. It compares the pairing relationships between clustering labels and true labels, eliminating the influence of random assignments, and provides a standardized score. The calculation formula is:


ARI=∑ij (CiCj)-[(CiCj)⋅(CjCi)]12(∑i (Ci2)+∑j (Cj2))

where (CiCj) represents the number of elements shared between the true cluster $C_{i}$ and the clustering partition $C_{j}$, and (Ci2) denotes the combination count. The ARI score ranges from [-1, 1], with higher values indicating clustering results closer to the true partition.

The normalized mutual information (NMI) is an information-theoretic metric that measures the mutual information between clustering partitions and true partitions. The calculation formula is:


$$\mathrm{NMI}=\frac{I(C,\;K)}{\sqrt{H(C)\cdot H(K)}}$$

where I(C, K) is the mutual information between clustering labels C and true labels K; H(C) and H(K) are the entropies of clustering labels and true labels, respectively, calculated as: $H(X)=-\sum_{x}\;P(x)\log P(x)$. The NMI score ranges from [0, 1], with values closer to 1 indicating better alignment between clustering results and true partitions.

The Accuracy (ACC) measures the proportion of clustering labels that match the true labels. The calculation formula is:


$$\mathrm{ACC}=\underset{\pi \in \Pi }{\max}\;\frac{\sum_{i=1}^{n}\;\delta (c_{i},\pi (k_{i}))}{n}$$

where Π is the set of all possible permutations (matches), $c_{i}$ represents clustering labels, $k_{i}$ represents true labels, and $\delta (c_{i},\pi (k_{i}))$ is an indicator function that is 1 if $c_{i}=\pi (k_{i})$ and 0 otherwise. The ACC score ranges from [0, 1], with higher values indicating more accurate clustering results. The Purity (PUR) evaluates the homogeneity of clusters by measuring the proportion of the majority class in each cluster. The calculation formula is:


$$\mathrm{PUR}=\frac{1}{n}\sum_{j=1}^{k}\;\underset{j}{\max}\;|\omega_{j}\cap c_{i}|$$

where n is the total number of samples, k is the number of clusters, $\omega_{j}$ represents the true class labels, and $c_{i}$ represents clustering labels. The PUR score ranges from [0, 1], with higher values indicating more accurate clustering results.cASW (cell-type average silhouette width) is a metric for evaluating the separation effectiveness of cell types after single-cell data integration. It quantifies the accuracy of cell type annotations by calculating the difference between the compactness within cell type clusters (intra-cluster similarity) and the separation between clusters (inter-cluster dissimilarity). The calculation is defined as:


$$\mathrm{cASW}=\;\frac{1}{n}\sum_{i=1}^{n}s(i),\;s(i)=\frac{b(i)-a(i)}{\max\{a(i),b(i)\}}$$

Here, a(i) represents the average distance from cell i to all other cells within its own cluster (intra-cluster distance), and b(i) represents the average distance from cell i to all cells in its nearest neighboring cluster (inter-cluster distance). cASW is the mean value of s(i) across all cells. cASW closer to 1 indicates clearer separation of cell types (tight clusters with good separation).cLISI (cell-type label local inverse Simpson’s index) quantifies the diversity of cell types within local neighborhoods in single-cell data. Effective cell type separation should result in each cell’s local neighborhood being predominantly composed of cells of the same type. In this study, a lower cLISI value indicates superior cell type separation, reflecting better preservation of biological variation. To maintain consistency with the evaluation standards, we applied the same linear transformation to cLISI as described in reference (Hu *et al.* 2024).

ARI, NMI, ACC, and PUR are used to assess the accuracy of cell clustering by the model. cLISI and cASW are used to evaluate the model’s capability to preserve biological specificity. We computed the mean of ARI, NMI, ACC, and PUR to assess the model’s overall clustering capability: cluster_avg=(ARI+NMI+ACC+PUR)/4. The mean values of iLISI and cASW are taken to evaluate the model’s comprehensive ability to retain biological specificity: bio_avg=(iLISI+cASW)/2. Take the average of all indicators to evaluate the comprehensive performance of the model on all indicators: performance = 1/6 (ARI + NMI + ACC + PUR + iLISI + cASW).

### 2.9 Experimental environment and parameter configuration

The algorithm is implemented in an environment with Python 3.8 and PyTorch (version 1.10.1 + cu111). The encoder structure of scECDA is configured as [input_dim, 500, 500, 2000, hidden], and the decoder structure is [hidden, 2000, 500, 500, input_dim], where input_dim represents the input data dimension and hidden denotes the latent feature dimension, more details are shown as Supplementary Table S3, available as supplementary data at *Bioinformatics* online. During the training phase, scECDA is trained in two parts: first, pre-training of the encoder and decoder, followed by end-to-end training of the entire network. The batch size is set to 256, and the number of classes must be specified by the user to complete model training. In terms of hardware configuration, the system uses CentOS 7 (kernel version 3.10.0–1160.95.1.el7.x86_64) and an NVIDIA A100 80GB PCIe GPU, with CUDA version 11.2.

## 3 Results

### 3.1 Evaluation of model clustering performance on datasets of different qualities and scales

To evaluate the clustering performance of the model on datasets with varying qualities and scales, we selected other eight datasets apart from SHARE_Mus_skin_filtered (as shown in Supplementary Table S1, available as supplementary data at *Bioinformatics* online). Among these datasets, CITE_PBMC_Inhouse and CITE_PBMC10x contain fewer cell types, whereas CITE_BMNC include a larger number of cell types. SNARE_Mus_Cortex, SHARE_Mus_Brain, and 10x Multiome_PBMC10x exhibit higher sparsity, while CITE _PBMC_Inhouse and Tea_PBMC represent small-scale datasets due to their limited cell numbers. In contrast, CITE_BMNC and 10x Multiome _BMMC are considered large-scale datasets given their relatively large cell numbers. The diverse characteristics of these datasets make them suitable for comprehensively comparing and analyzing the performance of different methods. To ensure fairness in the comparison of different methods, we uniformly specified the number of clusters for all methods to match the actual number of cell types in the datasets.

**Figure 2.**
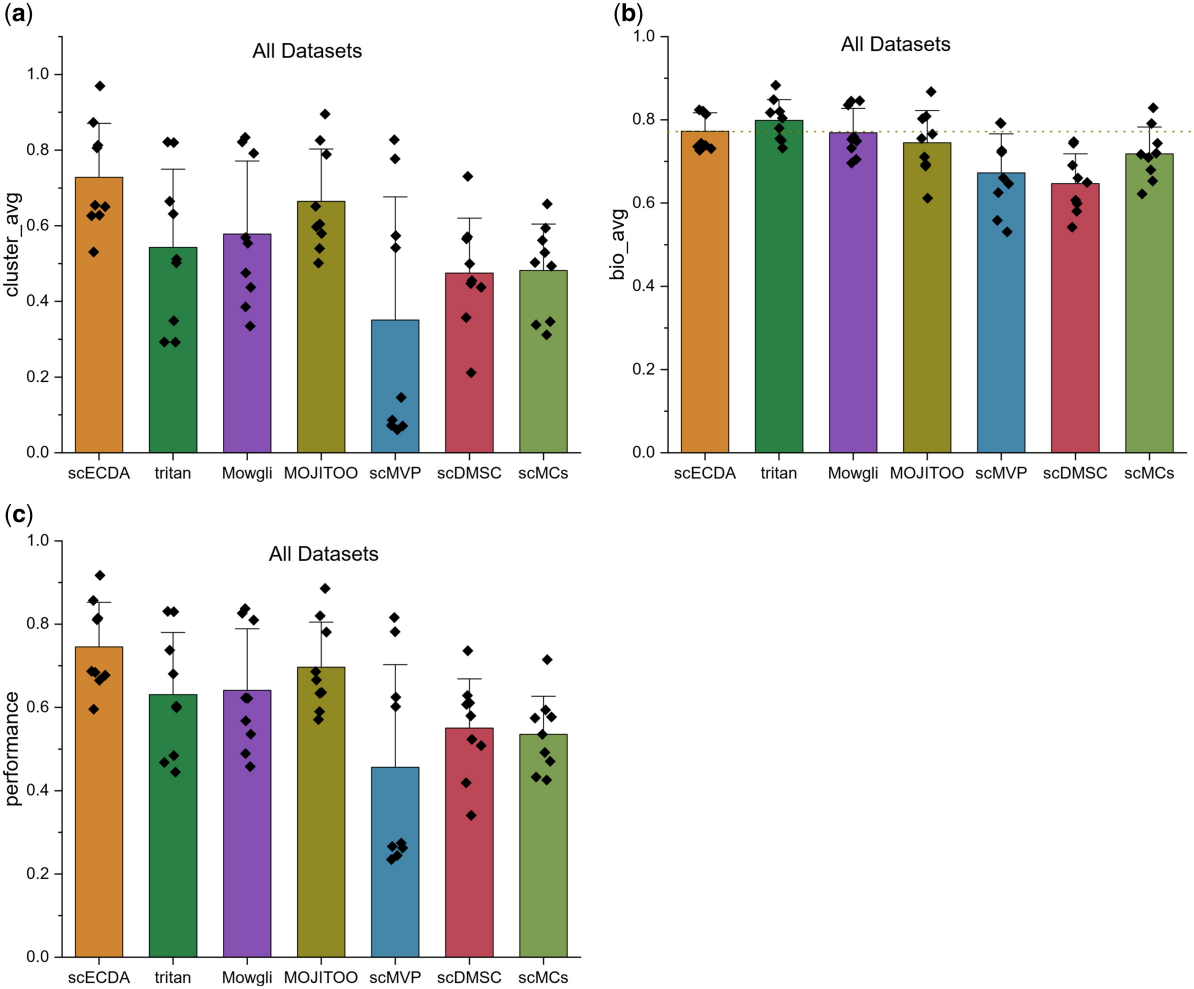
(a), (b), and (c) respectively represent the clustering accuracy, biological variance conservation and overall performance of different integration omics data methods on (RNA, ATAC), (RNA, ADT), and all datasets, where $\mathrm{performance}=\frac{1}{6}(\mathrm{ARI}+\mathrm{NMI}+\mathrm{ACC}+\mathrm{PUR}+\mathrm{iLISI}+\mathrm{cASW})$.

The results (Fig. 2) demonstrate that scECDA achieves the highest average clustering accuracy across eight datasets, ranks second in preserving biological variance, and exhibits the best overall performance. Further analysis (Supplementary Fig. S10, available as supplementary data at *Bioinformatics* online) reveals that scECDA performs exceptionally well on both RNA + ATAC and RNA + ADT datasets. This study aims to investigate the influence of specific omics data types on clustering outcomes and quantify their contributions using the K-means algorithm. Specifically, higher clustering accuracy for a particular omics data type indicates a greater contribution to the clustering results, while lower accuracy suggests a weaker contribution. As illustrated in Supplementary Figs S1a and S11, available as supplementary data at *Bioinformatics* online, the RNA data in the SHARE_Mus_Brain dataset significantly contributes to clustering (K-means_RNA: cluster_avg = 0.5824), whereas ATAC data shows a minimal contribution (K-means_ATAC: cluster_avg = 0.106). A similar pattern is observed in the SNARE_Mus_Cortex dataset (Supplementary Figs S2a and S12, available as supplementary data at *Bioinformatics* online). In this scenario, only scECDA surpasses K-means_RNA in average clustering accuracy. In contrast, other methods are more susceptible to data noise, particularly scMVP and scDMSC, which exhibit the most pronounced decline in clustering accuracy compared to K-means_RNA. Only scECDA, TriTan, and Mowgli outperform K-means_RNA on average (Supplementary Fig. S2, available as supplementary data at *Bioinformatics* online). Notably, in cases where ATAC contributes minimally to clustering, scECDA achieves the highest average clustering accuracy, demonstrating its superior robustness. This advantage stems from scECDA's feature fusion strategy, which effectively distinguishes true biological signals from sequencing noise, thereby mitigating noise interference in clustering.

Moreover, scECDA consistently achieves the highest average clustering accuracy across diverse scenarios, including: large-scale datasets, small-scale datasets, datasets with numerous cell types, datasets with fewer cell types, datasets with high sparsity. These findings further confirm scECDA’s strong robustness. Additionally, we observed that all methods perform better on ATAC datasets with higher clustering contributions compared to those with lower contributions (Supplementary Figs S1–S2 versus S3–S4, available as supplementary data at *Bioinformatics* online). Moreover, methods generally exhibit superior overall performance on RNA + ADT datasets than on RNA + ATAC datasets (Supplementary Figs S1–S5 versus S6–S9, available as supplementary data at *Bioinformatics* online).

### 3.2 Evaluation of model clustering performance on single-omics and multi-omics datasets

**Figure 3.**
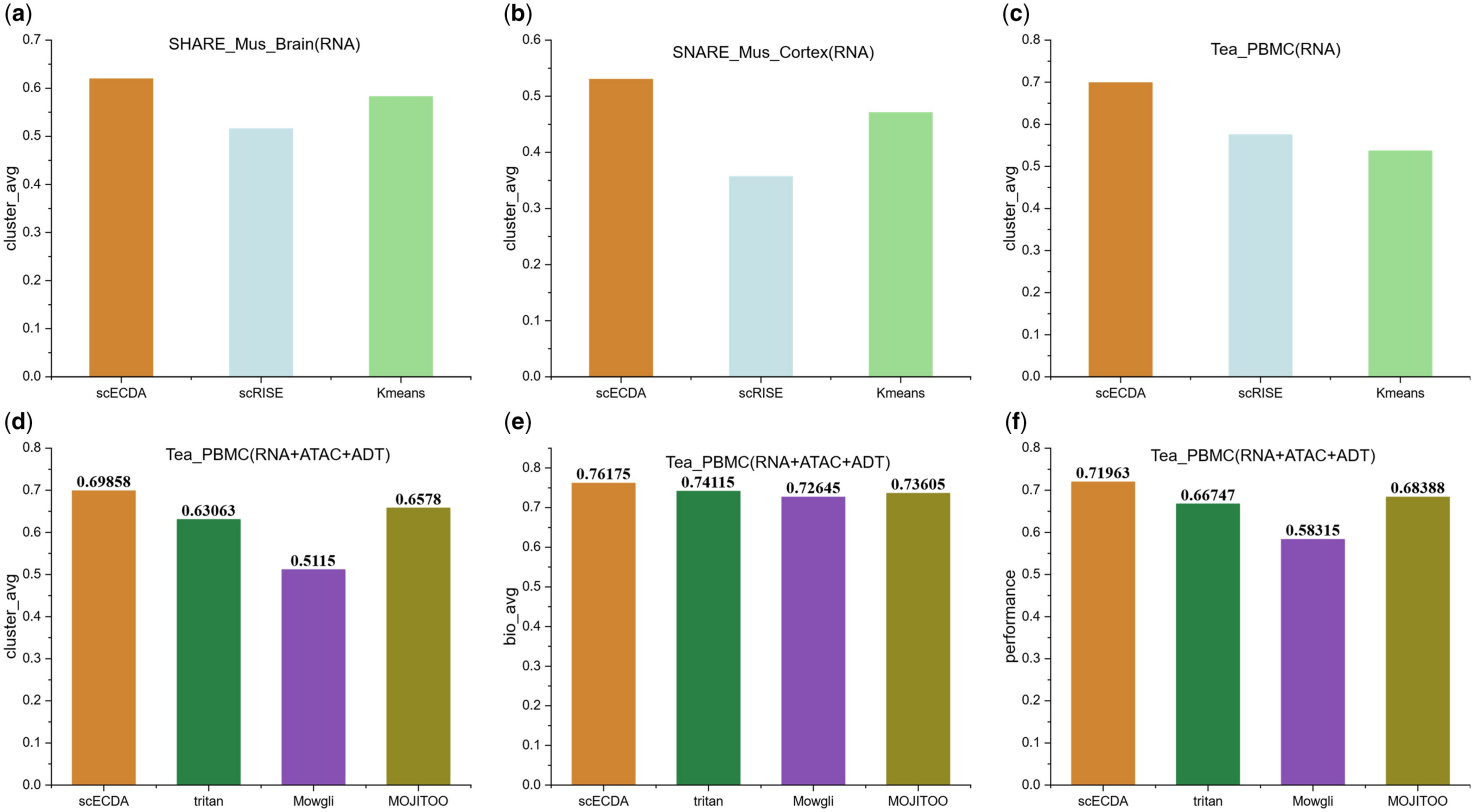
Clustering performance comparison of different methods on (a) RNA-seq data from SHARE_Mus_Brain, (b) RNA-seq data from SNARE_Mus_Cortex, (c) RNA-seq data from Tea_PBMC, and (d), (e), (f) multi-omics Tea_PBMC data.

scECDA is not only capable of integrating two single-cell omics datasets but can also process three-omics data. Additionally, it can perform clustering analysis when only one single-cell omics dataset is available. To evaluate the performance of the scECDA method on both single-omic and multi-omics datasets, this study employed the same metrics—cluster_avg and bio_avg. We selected the RNA data from the SNARE_Mus_Cortex dataset, the RNA data from the SHARE_Mus_Brain dataset, the RNA data from the Tea_PBMC dataset, and the three-omics data from the Tea_PBMC dataset as test datasets. Figure 3a–c illustrate the evaluation results of scECDA on single-omics datasets. It can be observed that scECDA achieves the highest scores in clustering accuracy across these three datasets. Figure 3d–f present the evaluation results of scECDA on the Tea_PBMC dataset, which includes three omics data. It is evident that scECDA attains the highest scores in both clustering accuracy and the ability to preserve biological variance. Through the aforementioned comparative analysis, it is clear that scECDA outperforms other methods in clustering tasks, whether dealing with single-omics data or multi-omics data comprising three omics.

### 3.3 Evaluation of model clustering performance across different types of omics data

Ideally, the higher the quality and variety of omics data available, the richer the information provided and the more accurate the clustering results. Therefore, a robust model should effectively integrate information from various omics datasets, thereby enhancing the accuracy of clustering outcomes. To investigate whether scECDA can effectively integrate information from multiple omics datasets, we utilized the tea dataset for experimentation. The clustering results of K-means on RNA and ATAC data, as shown in Fig. 3c, indicate that the quality of these datasets is relatively high, aligning closely with ideal conditions. In this experiment, the tea dataset was processed in the following configurations: paired as (RNA, ATAC), (RNA, ADT), and not split (RNA, ATAC, ADT).

**Figure 4.**
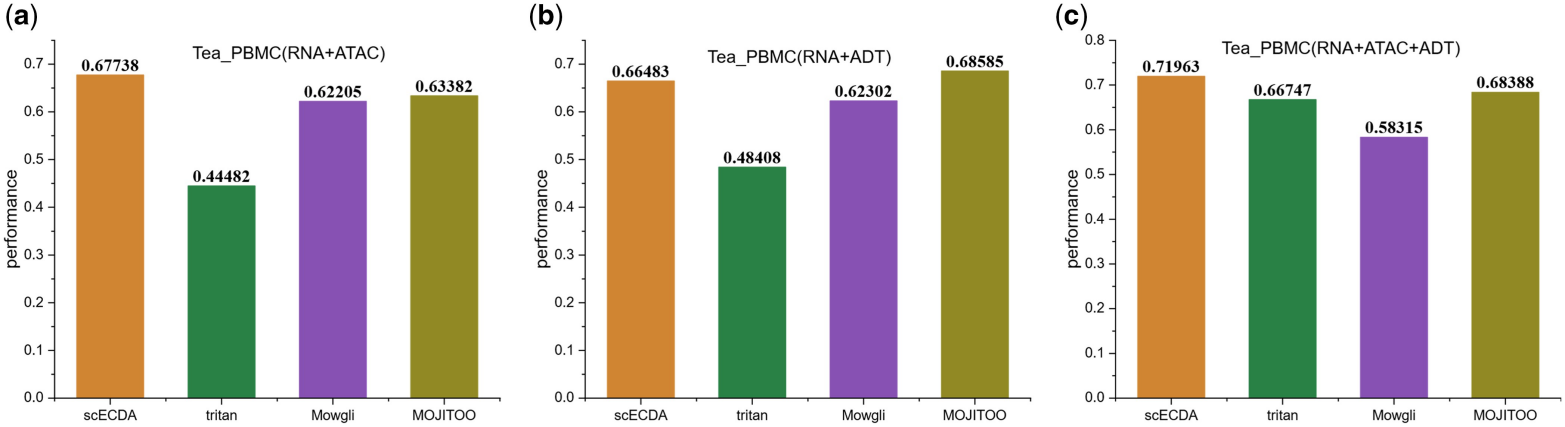
Comparison of model overall performance across different types of omics data.

The experimental results in Fig. 4 demonstrate the performance differences of various methods in multi-omics data fusion. scECDA and TriTan exhibit significantly higher clustering scores when integrating three types of omics data compared to using only two types of omics data. However, Mowgli shows a decrease in clustering accuracy as the number of omics data increases, which is further supported by Supplementary Fig. S13, available as supplementary data at *Bioinformatics* online. This phenomenon indicates a limitation of Mowgli in integrating more types of omics data. MOJITOO performs worse on the RNA + ADT dataset than in the scenario with three omics data. In comparison, scECDA is capable of effectively integrating heterogeneous information from different omics platforms.

### 3.4 Evaluation of model clustering performance across multiple batch datasets

**Figure 5.**
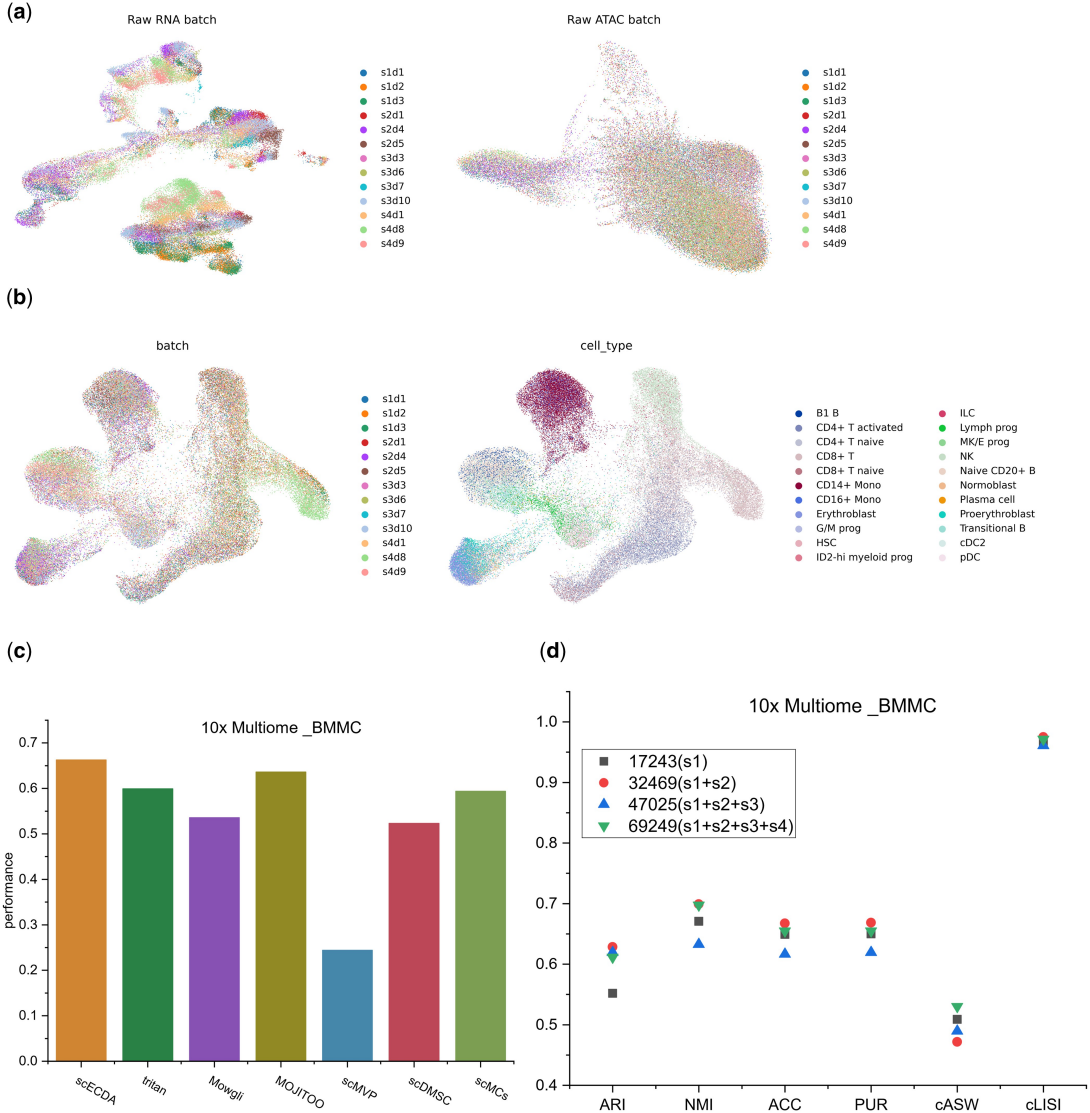
(a) Represents the distribution of data from different batches in the raw data; (b) Shows the distribution of batch data after batch effect removal by scECDA; (c) evaluates the clustering results of different methods on the 10× Multiome _BMMC dataset; (d) investigates the impact of the number of batches on scECDA clustering results, where s1 (s1d1, s1d2, s1d3), s2 (s2d1, s2d4, s2d5), s3 (s3d3, s3d6, s3d7, s3d10), and s4 (s4d1, s4d8, s4d9) represent four large batches, and “all” refers to the combination of s1 + s2 + s3 + s4.

Batch effects refer to technical variations introduced during the processing and measurement of experimental samples across different batches due to differences in factors such as time, operators, reagents, and instruments. These technical variations are unrelated to biological variations and may obscure or confound true biological differences. In single-cell data analysis, particularly when integrating sequencing data from different batches, batch effects can lead to biases in the analysis results. Therefore, it is essential to correct for batch effects before conducting the analysis to minimize batch-to-batch differences and ensure the accuracy and reliability of the data. To investigate the ability of scECDA to integrate data and perform clustering on datasets with multiple batches, we selected the BMMC dataset (containing 12 small batches across 3 large batches) to validate its performance. Figure 5a and b demonstrate the changes in data before and after integration. It can be observed that cells with similar features are effectively clustered together, and batch effects are removed. The quality of data integration is shown in Fig. 5c, where scECDA achieves the highest comprehensive score. Figure 5d displays the impact of multi-batch data combination on clustering results, with numerical fluctuations within 0.05, indicating good stability of scECDA. When integrating multi-batch data, scECDA can effectively mitigate batch effects, showing small fluctuations across various indicators and stable performance.

### 3.5 Discovery of biological biomarkers

**Figure 6.**
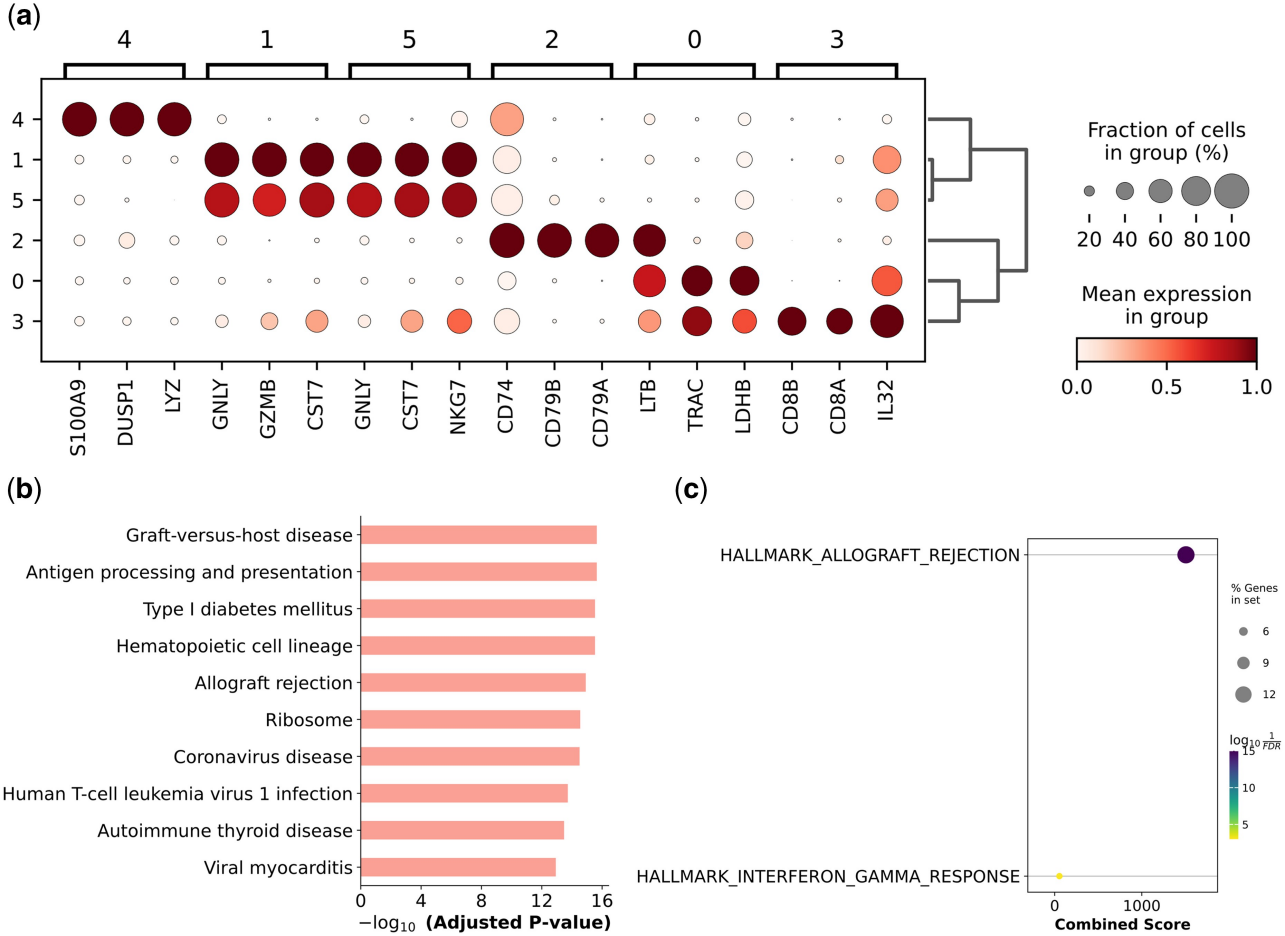
Identify the top three differentially expressed genes from the CITE_PBMC_Inhouse dataset and plot their gene expression distribution (a); perform gene enrichment analysis (b) and (c).

Biomarkers play a crucial role in guiding cell clustering, particularly in the analysis of high-throughput single-cell data, as their expression patterns can effectively reflect cellular heterogeneity and provide important insights into gene regulatory mechanisms. To validate the accuracy of single-cell analysis methods in cell clustering, this experiment employed non-parametric statistical methods (such as the Wilcoxon rank-sum test) to screen for the top three most significantly differentially expressed features (including genes, surface proteins, and open chromatin regions) in each predicted cluster. These features were then validated for their specificity using authoritative databases such as GeneCards (Safran *et al.* 2010) and GenBank (Benson *et al.* 2018). The biomarkers identified by scECDA on the CITE_PBMC_Inhouse dataset are shown in Fig. 6a and Supplementary Fig. S14, available as supplementary data at *Bioinformatics* online, with detailed descriptions provided in the Supplementary File (P24), available as supplementary data at *Bioinformatics* online.

Single-cell omics data are complementary, enabling a more comprehensive analysis of cellular heterogeneity. By integrating transcriptomic (transcriptomics) and surface proteomic (surface proteomics) data, we can more accurately identify and distinguish different cellular subpopulations. In the CITE_PBMC_Inhouse dataset, CD8+ T cells and CD4+ T cells share similar functions and are challenging to distinguish in two-dimensional visualization (e.g. Supplementary Fig. S14, available as supplementary data at *Bioinformatics* online). In such cases, biomarkers are essential. According to the differential gene expression distribution (Supplementary Fig. S14, available as supplementary data at *Bioinformatics* online), CD8A and CD8B genes are almost exclusively highly expressed in CD8+ T cells. This is because CD8A promotes the survival and differentiation of activated lymphocytes into memory CD8+ T cells (Littman *et al.* 1985, Nakayama *et al.* 1989), while CD8B plays a critical role in the thymic selection of CD8+ T cells (Norment and Littman 1988, Arcaro *et al.* 2001). According to the differential protein expression distribution (Supplementary Fig. S15, available as supplementary data at *Bioinformatics* online), CD4 is specifically expressed in CD4+ T cells. CD4 gene expression is strictly regulated by specific transcription factors and gene regulatory networks, ensuring that CD4 is expressed only in particular cell types, such as helper T cells and monocytes. Transcription factors like T-cell factor 1 (TCF-1) and GATA3 are activated in helper T cells, promoting CD4 gene expression (Littman *et al.* 1988, Maddon *et al.* 1985). In other immune cell types (e.g. B cells, CD8+ T cells), these specific regulatory factors are typically inactive, leading to the suppression of CD4 gene expression (Hodge *et al.* 1991, Ansari-Lari *et al.* 1996). Therefore, these biomarkers, with their specific expression patterns, can be used to distinguish CD8+ T cells from CD4+ T cells.

To further explore the enrichment significance of gene sets in different biological pathways, this study selected the top 20 differentially expressed genes in each cluster, took their intersection, formed gene sets, and performed gene enrichment analysis (Gene Ontology, GO). As shown in Fig. 6b, the gene sets were highly enriched in pathways related to “immune-related diseases (autoimmune diseases, transplant immunopathology), immune activation processes (antigen presentation), and viral infection pathology”, with extremely high statistical significance for these enrichments. The study classified the enrichment terms into three categories: (i) Immune-related diseases and processes: Graft-versus-host disease, Antigen processing and presentation, Type I diabetes mellitus, Autoimmune thyroid disease, Allograft rejection; (ii) Infectious diseases: Coronavirus disease, Human T-cell leukemia virus 1 infection, Viral myocarditis; (iii) Cellular components and basic functions: Ribosome, Hematopoietic cell lineage. Among them, graft-versus-host disease and allograft rejection jointly reflect the enrichment trend of bidirectional damage in transplant immunity, while antigen processing and presentation is a core step of immune response (dendritic cells and others capture, process antigens, and present them to T cells, initiating adaptive immunity), indicating that the gene sets are highly enriched in adaptive immune activation. Meanwhile, Fig. 6c also shows that allograft rejection is a core enriched pathway.

### 3.6 Cluster-specific motif recovery

This study further explores the application value of scECDA in identifying cell type-specific transcription factor binding motifs, which plays a key role in deciphering gene regulatory networks under specific biological contexts. Using the human peripheral blood mononuclear cell dataset 10x Multiome_PBMC10x, we performed whole-cell clustering with the scEDCA model and combined it with the chromVAR algorithm to screen for cell type-specific enriched motifs from the JASPAR database and quantify their enrichment scores. The analysis results in Supplementary Fig. S16, available as supplementary data at *Bioinformatics* online show that MA0497.1 (corresponding to transcription factor MEF2C), MA0496.3 (MAFK), MA0017.2 (NR2F1), and MA0687.1 (SPIC) exhibit significant enrichment in clusters 13 and 5; MA1491.1 (GLI3) shows high enrichment in clusters 6, 10, and 13. The sequences of transcription factor (TF) binding motifs are shown in Supplementary Figs S17 and S18, available as supplementary data at *Bioinformatics* online.

Combining cell type annotations and transcription factor functional annotations, we can provide mechanistic explanations for the above enrichment phenomena: cluster 13 corresponds to CD14 monocytes (CD14 Mono) and circulating dendritic cell precursors (CDC), while cluster 5 corresponds to CD16 monocytes (CD16 Mono); according to GeneCards, MEF2C, as a core regulatory factor in immune cell (monocyte, T cell, etc.) lineages, deeply participates in the epigenetic regulation of cell differentiation, survival, and inflammatory pathways; SPIC directly mediates chromatin state remodeling in processes such as monocyte differentiation, phagocytic function, and cytokine secretion. The correlation between cell types and transcription factor functions provides physiological evidence for motif enrichment patterns.

### 3.7 Trajectory inference

**Figure 7.**
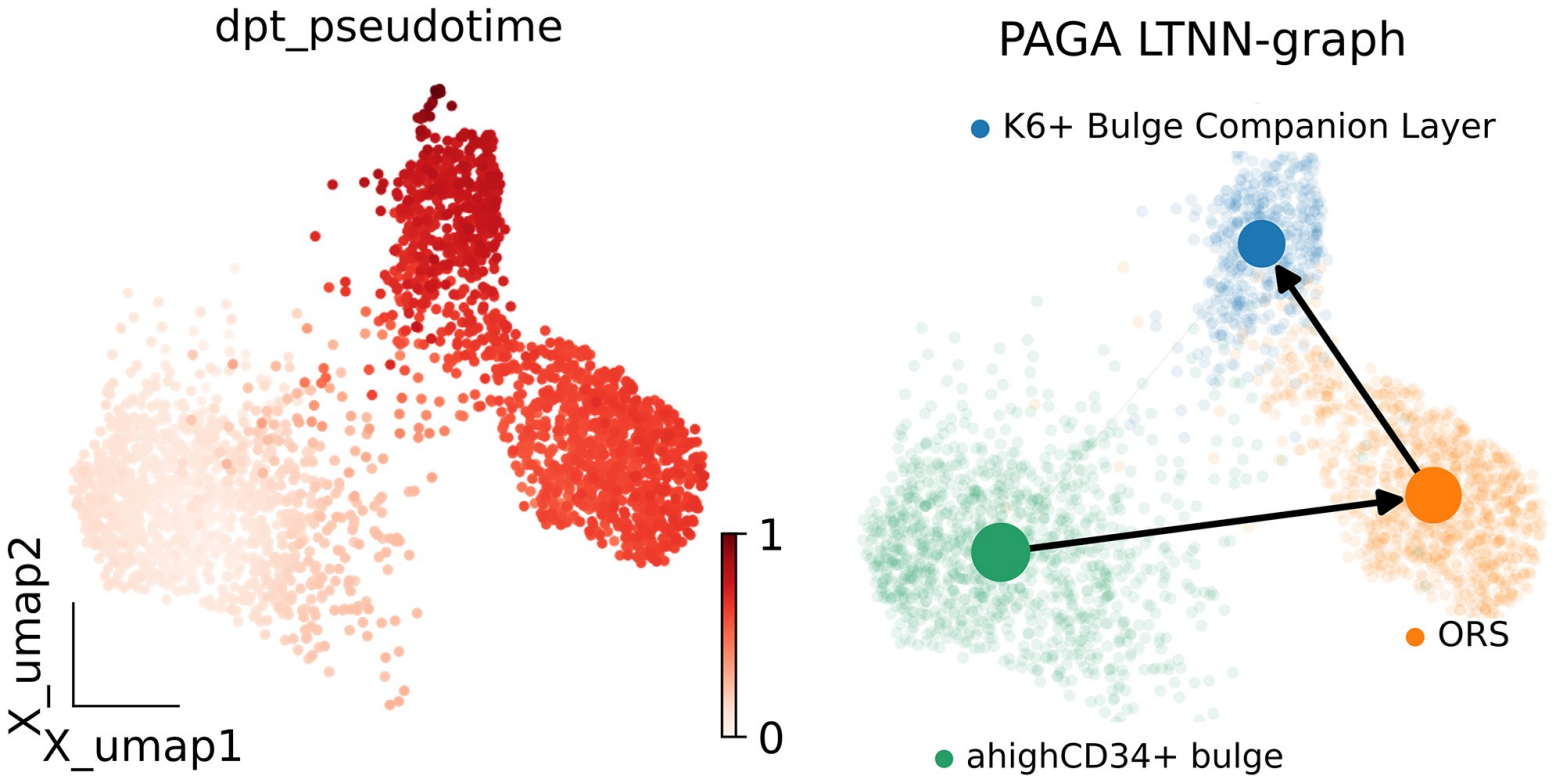
Inferring cell trajectories using potential features obtained by integrating omics data with scECDA.

In this study, we employed the PAGA (Wolf *et al.* 2019) method to analyze the differentiation trajectories of mouse skin cells, with the cell data sourced from the SHARE_Mus_skin dataset. We subjected the original dataset to rigorous filtering, retaining only cells classified as K6^+^ Bulge Companion Layer, ORS, and $\alpha^{\mathrm{high}}$CD34^+^ bulge, thereby generating the SHARE_Mus_skin_filtered dataset. Subsequently, we harnessed the scEDCA model to extract and integrate the fused features of the data. The differentiation trajectory inferred by PAGA was identified as $\alpha^{\mathrm{high}}$CD34^+^ bulge -> ORS -> K6^+^ bulge companion layer, as depicted in Fig. 7. Notably, this trajectory aligns with one of the cell differentiation and development pathways reported in the SHARE-seq (Ma *et al.* 2020) literature ($\alpha^{\mathrm{high}}$CD34^+^ bulge -> ORS ->New bulge), further underscoring the effectiveness of scECDA in integrating omics data and facilitating the inference of cell trajectories. It has significant implications for the discovery of novel biological insights.

### 3.8 Ablation studies

To further investigate the individual impact of the proposed modules on the overall performance, we conducted comprehensive ablation studies. Specifically, we constructed five variants of scECDA and compared their performance on clustering tasks. These variants involved removing the contrastive learning module, the data augmentation module, and replacing the differential attention-based fusion module with a self-attention mechanism in various combinations. By analyzing the performance differences among these variants, we could evaluate the specific contributions of each module to the model’s performance.

The experimental results are shown in Supplementary Table S2, available as supplementary data at *Bioinformatics* online. First, by comparing the results of variants ①② and ③④, we observed that the differential attention-based fusion module significantly outperformed the self-attention mechanism. This indicates that the differential attention mechanism is more effective in integrating data from different omics, identifying more critical features within the data, and increasing their relative importance. Second, by comparing the results of variants ①③ and ②④, we found that employing contrastive learning during model training yielded better results. This suggests that contrastive learning helps the model learn more meaningful data representations, mapping the multi-omics data of the same cell closely together in the latent space while separating the multi-omics data of different cells. Finally, by comparing the results of variants ④⑤ and ③⑥, we discovered that incorporating the data augmentation module further enhanced the model’s clustering performance. This indicates that the positive and negative sample pairs created by the data augmentation module can assist contrastive learning during training to learn more meaningful features.

In summary, our ablation studies demonstrated the importance of each proposed module and their positive impact on the overall performance of the method.

### 3.9 Parameter analysis

**Figure 8.**
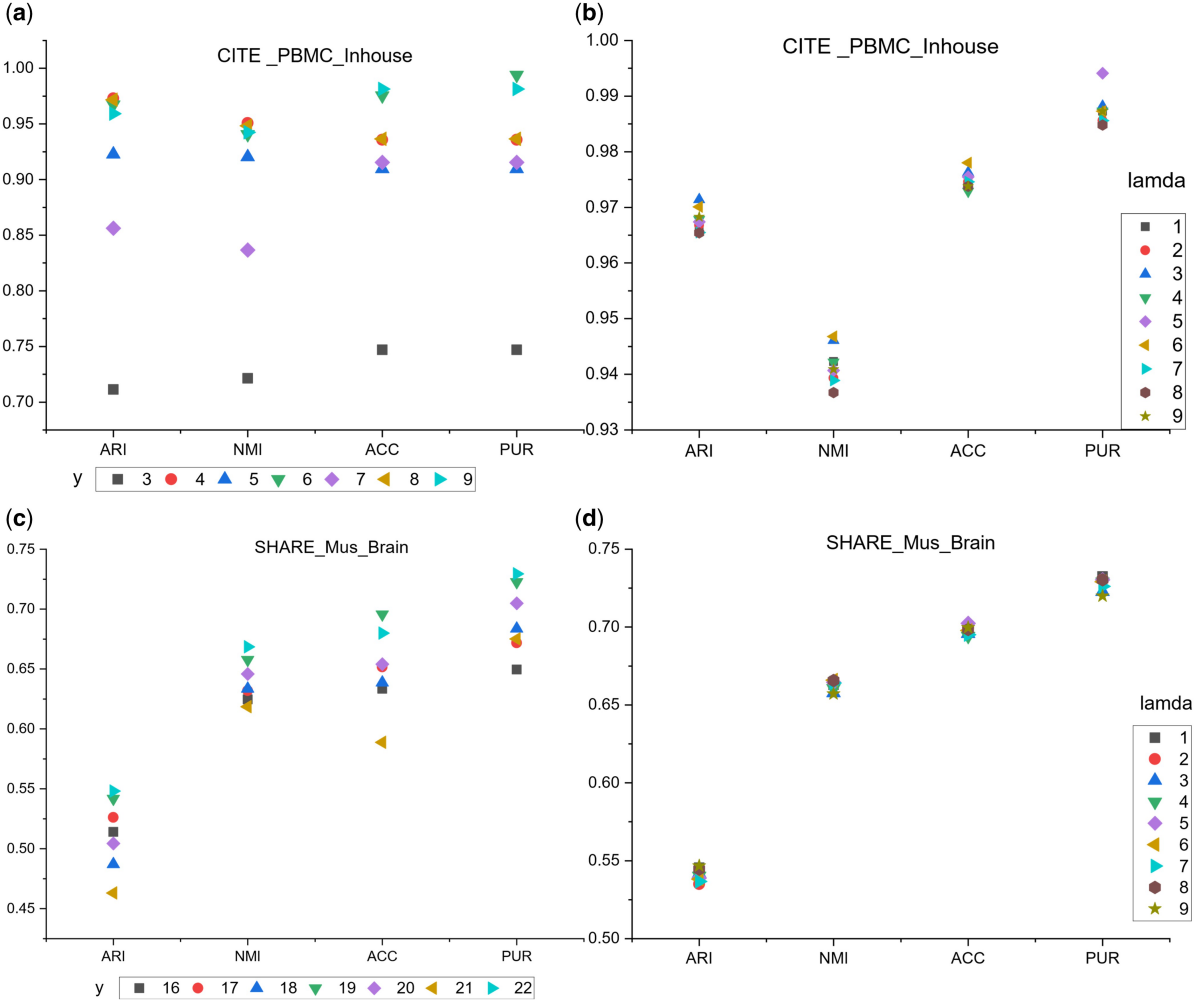
Effect of the values of y and $\lambda_{0}$ on the model. We conduct experiments on CITE_PBMC_Inhouse (a)(b) and SHARE_Mus_Brain (c)(d) dataset.

This section primarily discusses the impact of two hyperparameters, y and $\lambda_{0}$, on the clustering results in scECDA. Specifically, y represents the number of cell types, while $\lambda_{0}$ is used to initialize λ. The experimental results on the InHouse dataset are shown in Fig. 8a. Except for a significant drop in performance metrics when y=3, the clustering results remain relatively stable across other values of y. The optimal clustering results are achieved when y is set to the actual number of cell types in the dataset. Figure 8b illustrates the impact of varying $\lambda_{0}$ on the clustering results. The results indicate that regardless of the value of $\lambda_{0}$ within the range of 1 to 9, the performance of scECDA remains highly stable, with variations in metrics limited to approximately 0.01. Similar observations were made on the Mouse Brain dataset, as shown in Fig. 8c. Within the range y∈[16, 22], the clustering results exhibit minimal variation, achieving optimal performance when y matches the actual number of cell types. Figure 8d further confirms that changes in $\lambda_{0}$ have a negligible impact on clustering results, with performance variations of less than 0.02 across all metrics. Based on the above analysis, we conclude: (i) the value of $\lambda_{0}$ has minimal impact on model performance; (ii) for the selection of y, it is recommended to set it close to or equal to the actual number of cell types to achieve the best clustering results.

### 3.10 Model convergence analysis

This section mainly discusses the model’s convergence and whether the decrease in loss values can improve clustering accuracy (ACC). The specific results are shown in Supplementary Fig. S19, available as supplementary data at *Bioinformatics* online. Supplementary Fig. S19a, available as supplementary data at *Bioinformatics* online shows that during the pre-training stage, the loss value continuously decreases with increasing training epochs. Supplementary Figure S19b, available as supplementary data at *Bioinformatics* online shows that during the training stage, both the loss value and ACC metrics tend to stabilize with increasing training epochs. Based on the changes in the loss curve and the acc curve, for the majority of datasets, scECDA converges within 200 pre-training and training epochs, satisfying the stopping criteria.

## 4 Discussion

This study proposes a novel framework for integrating single-cell multi-omics data, termed scECDA, which leverages enhanced contrastive learning and a differential attention mechanism. The framework employs independently designed autoencoders to autonomously learn the feature distributions of each omics dataset. During the data integration process, an enhanced contrastive learning strategy is utilized to align features across different omics datasets. Additionally, the differential attention mechanism is incorporated to amplify critical biological signals, such as gene-specific expression patterns, while minimizing technical noise, including batch effects and sequencing errors. The model is flexible, capable of adapting to single-cell omics data generated by various technological platforms, and directly outputs integrated latent features along with end-to-end cell clustering results.

This study evaluates the performance of scECDA against eight existing mainstream methods across eight paired single-cell multi-omics datasets. The results demonstrate that scECDA achieves the best overall performance in most datasets, particularly excelling in handling datasets with high sparsity and large scales, where its clustering accuracy surpasses other methods significantly. This underscores the robust noise resistance of scECDA. Moreover, scECDA performs exceptionally well not only on two-omics data but also on single-omics and three-omics datasets, showcasing its versatility. To further investigate scECDA’s ability to utilize information from different types of omics data, the Tea_PBMC dataset was partitioned into two- and three-omics configurations. The results reveal that scECDA’s clustering accuracy on the three-omics data is significantly higher than on the two-omics data. Furthermore, validation experiments on multi-batch datasets confirm that the addition of batch data has minimal impact on scECDA’s clustering performance, with accuracy variations limited to within 0.05. Ablation studies further corroborate the effectiveness of the differential attention-based fusion module and the data augmentation module. Biological validation experiments successfully identified subtype-specific biomarkers, such as CD8A and CD8B, in the CITE _PBMC_Inhouse dataset, recovered cluster-specific motif and inferred cell trajectory, with results validated through gene databases and relevant literature, thereby confirming the biological significance of the method in deciphering cellular heterogeneity.

However, in the latent space obtained by integrating single-cell data using scECDA, cells within the same cluster are not distributed compactly enough. Moreover, the number of clusters needs to be specified when clustering. The current scECDA framework has yet to incorporate spatial transcriptomics, a critical dimension of single-cell data. Given the importance of spatial heterogeneity at single-cell resolution for cell type identification and functional analysis, the research team plans to develop algorithms integrating spatial information in future work. By establishing a “multi-omics-spatial” integrative analysis framework, scECDA aims not only to enhance clustering accuracy but also to provide comprehensive data support and theoretical foundations for precision medicine research.

## Supplementary Material

btaf443_Supplementary_Data

## Data Availability

The datasets downloaded from the GEO database include SNARE_Mus_Cortex (GSE126074), SHARE_Mus_Brain (GSE140203), 10x Multiome_BMMC (GSE194122), and CITE _PBMC_Inhouse (GSE148665). Additionally, datasets from other papers include CITE_BMNC (https://github.com/satijalab/seurat-data), 10x Multiome_PBMC10x (https://support.10xgenomics.com/single-cell-multiome-atac-gex/datasets/1.0.0/pbmc_granulocyte_sorted_10k), Tea_PBMC (https://github.com/PYangLab/Matilda/tree/main/data/TEAseq), and CITE _PBMC10x (https://github.com/jianghruc/scHoML). Source codes for the scEDCA python packages and the related scripts are available at (https://github.com/SuperheroBetter/scECDA).
