# Supplementary material for: Multi-omics single-cell data alignment and integration with enhanced contrastive learning and differential attention mechanism
Source: Bioinformatics. 2025 Aug 7;41(8):btaf443. doi: 10.1093/bioinformatics/btaf443 (PMC12543095; doi:10.1093/bioinformatics/btaf443)
Supplement: btaf443_Supplementary_Data [file btaf443_supplementary_data.pdf]

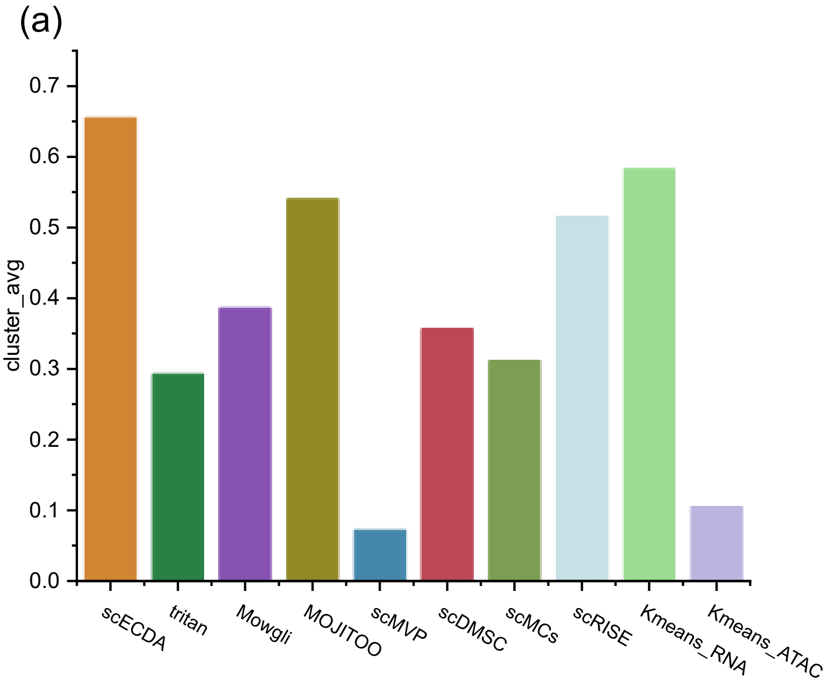

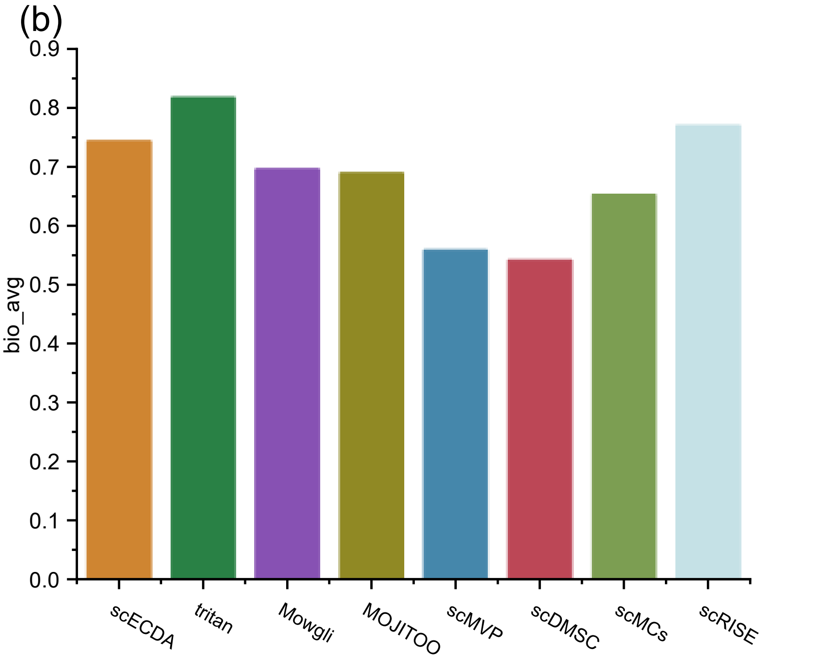

Fig S1. Evaluate the clustering accuracy and the ability to preserve biological specificity of different methods on the SHARE_Mus_Brain dataset.

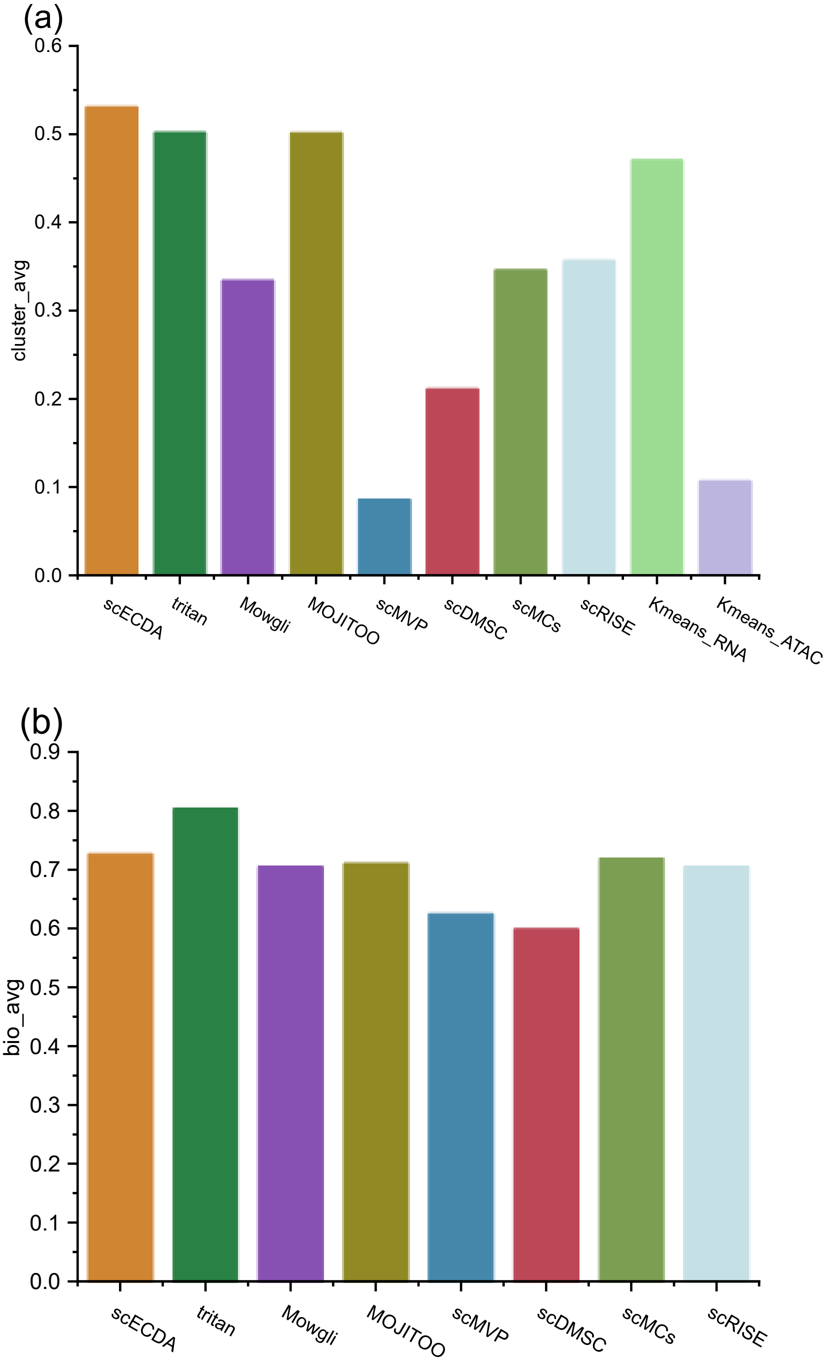

Fig S2. Evaluate the clustering accuracy and the ability to preserve biological specificity of different methods on the SNARE_Mus_Cortex dataset.

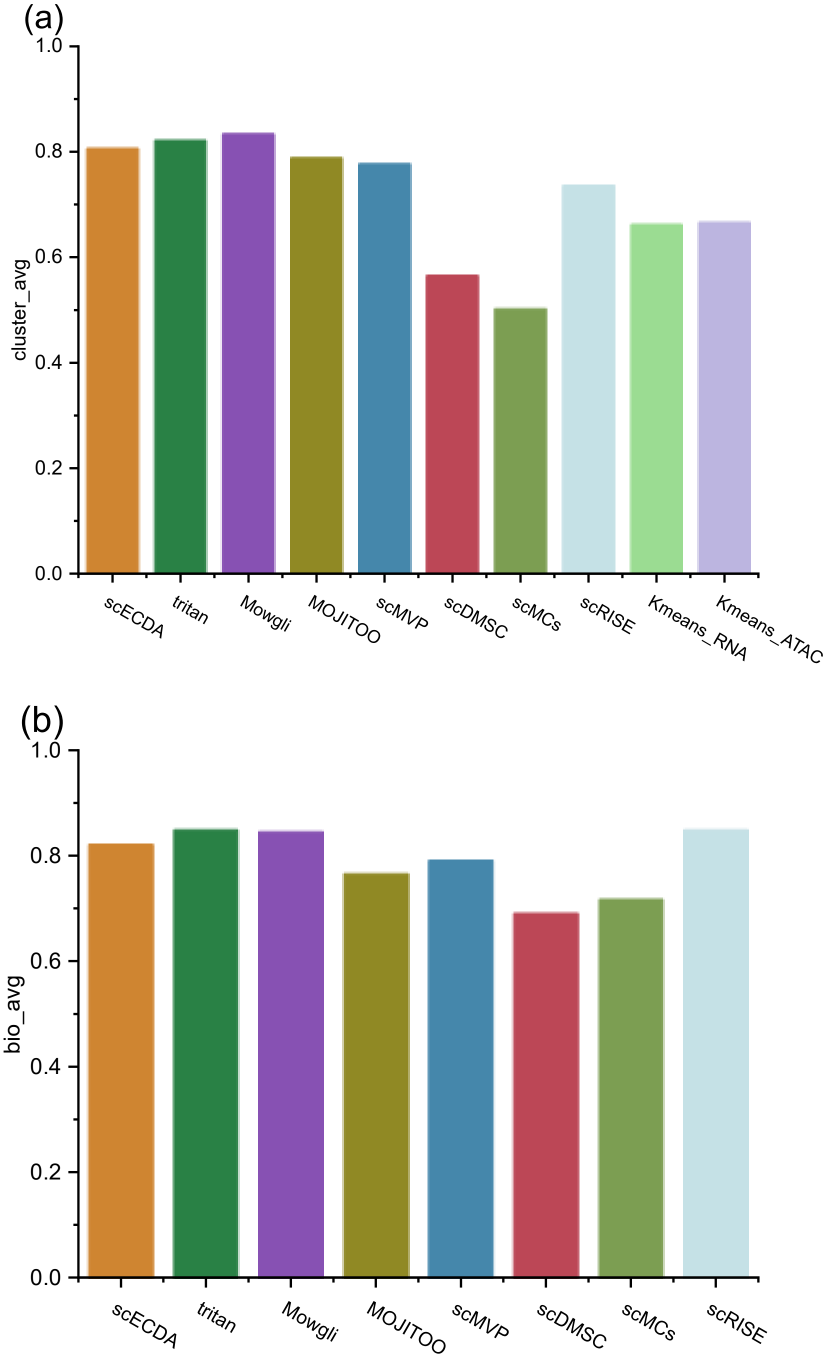

Fig S3. Evaluate the clustering accuracy and the ability to preserve biological specificity of different methods on the 10x Multiome_PBMC10x dataset.

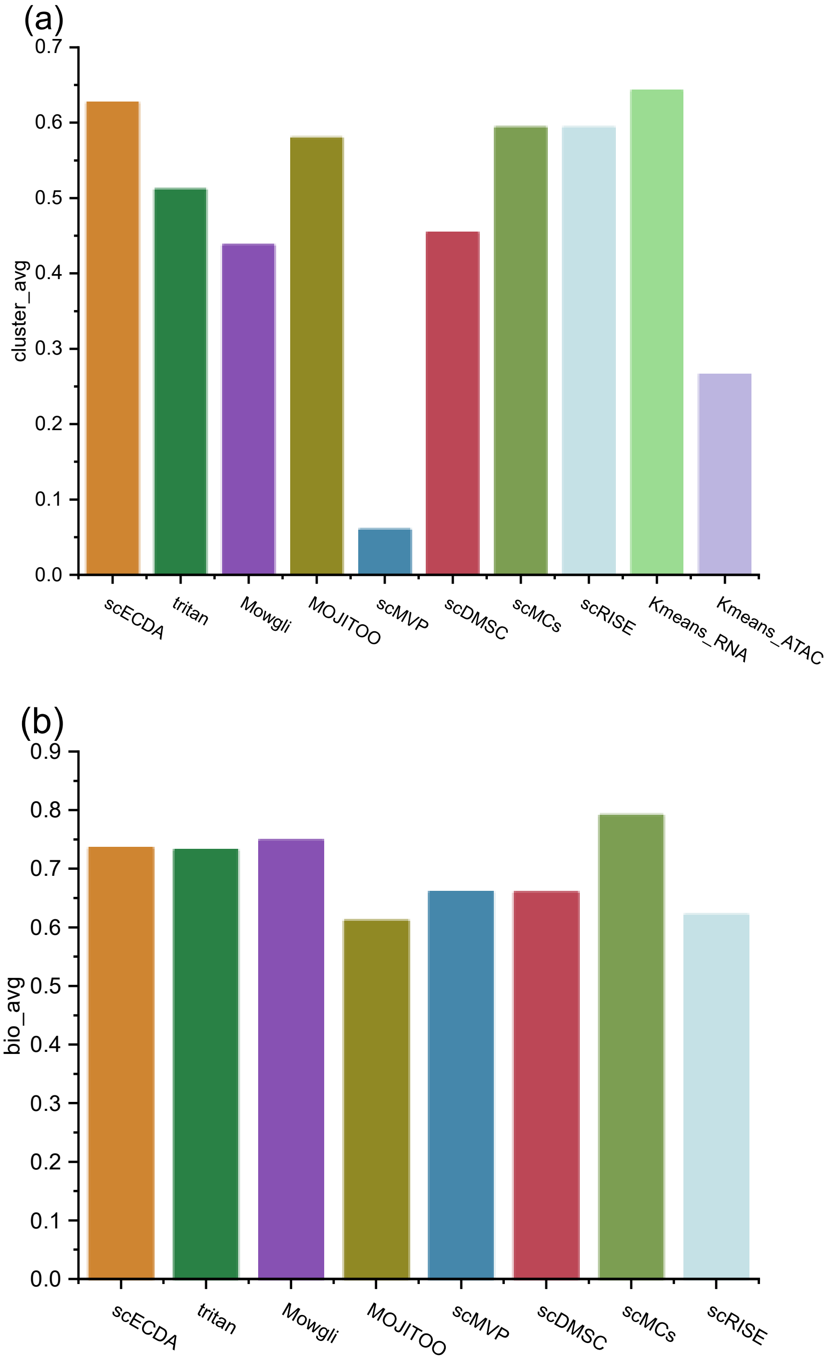

Fig S4. Evaluate the clustering accuracy and the ability to preserve biological specificity of different methods on the 10x Multiome _BMMC dataset.

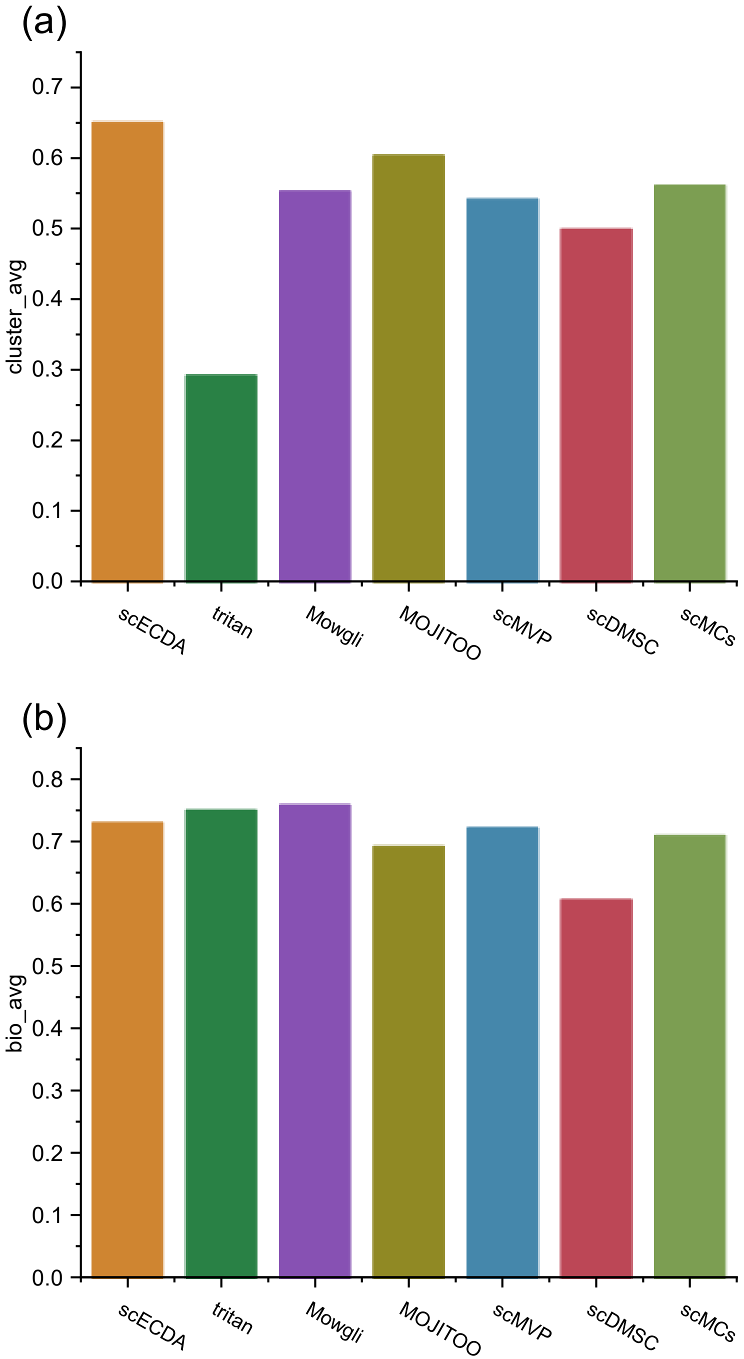

Fig S5. Evaluate the clustering accuracy and the ability to preserve biological specificity of different methods on the Tea_PBMC(RNA+ATAC) .

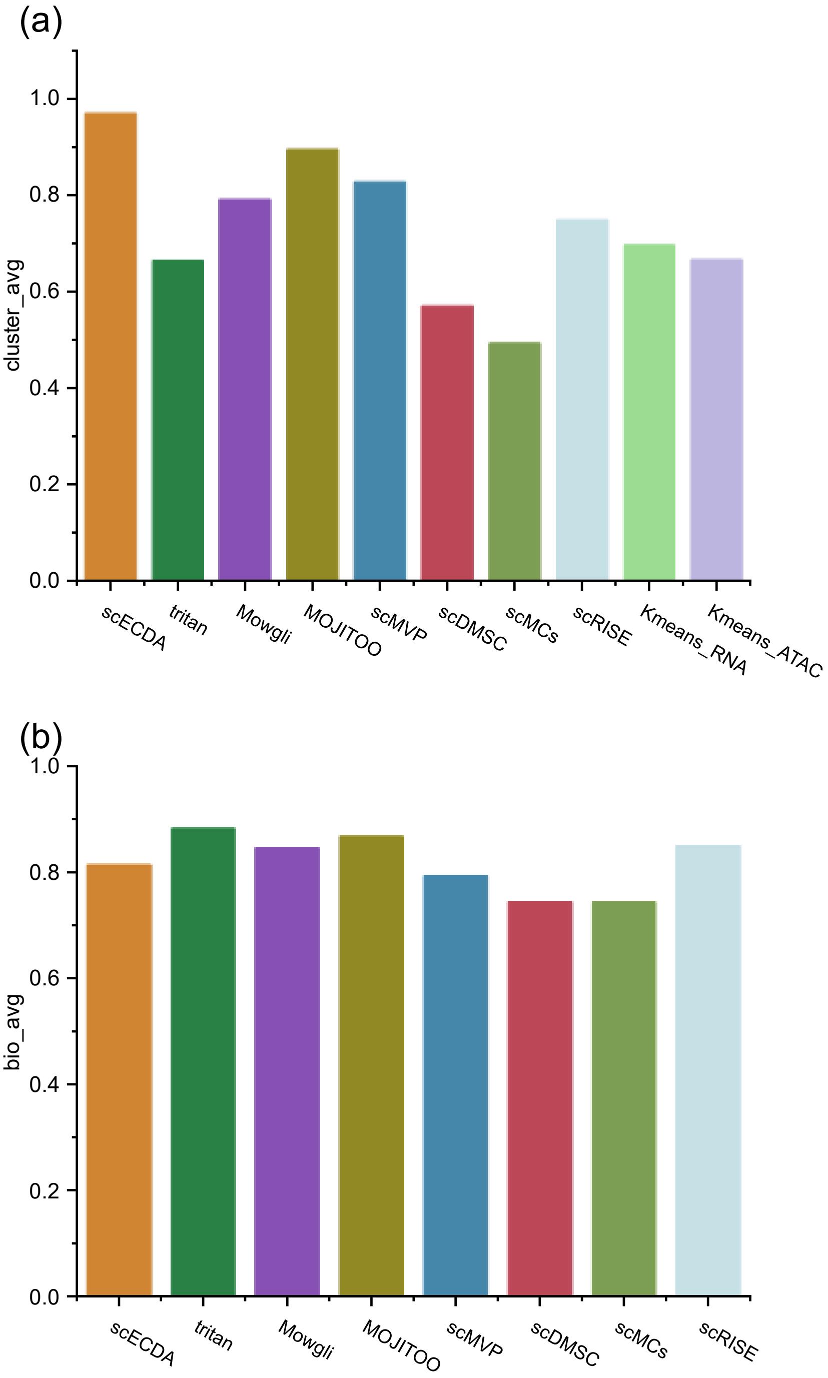

Fig S6. Evaluate the clustering accuracy and the ability to preserve biological specificity of different methods on the CITE _PBMC_Inhouse dataset.

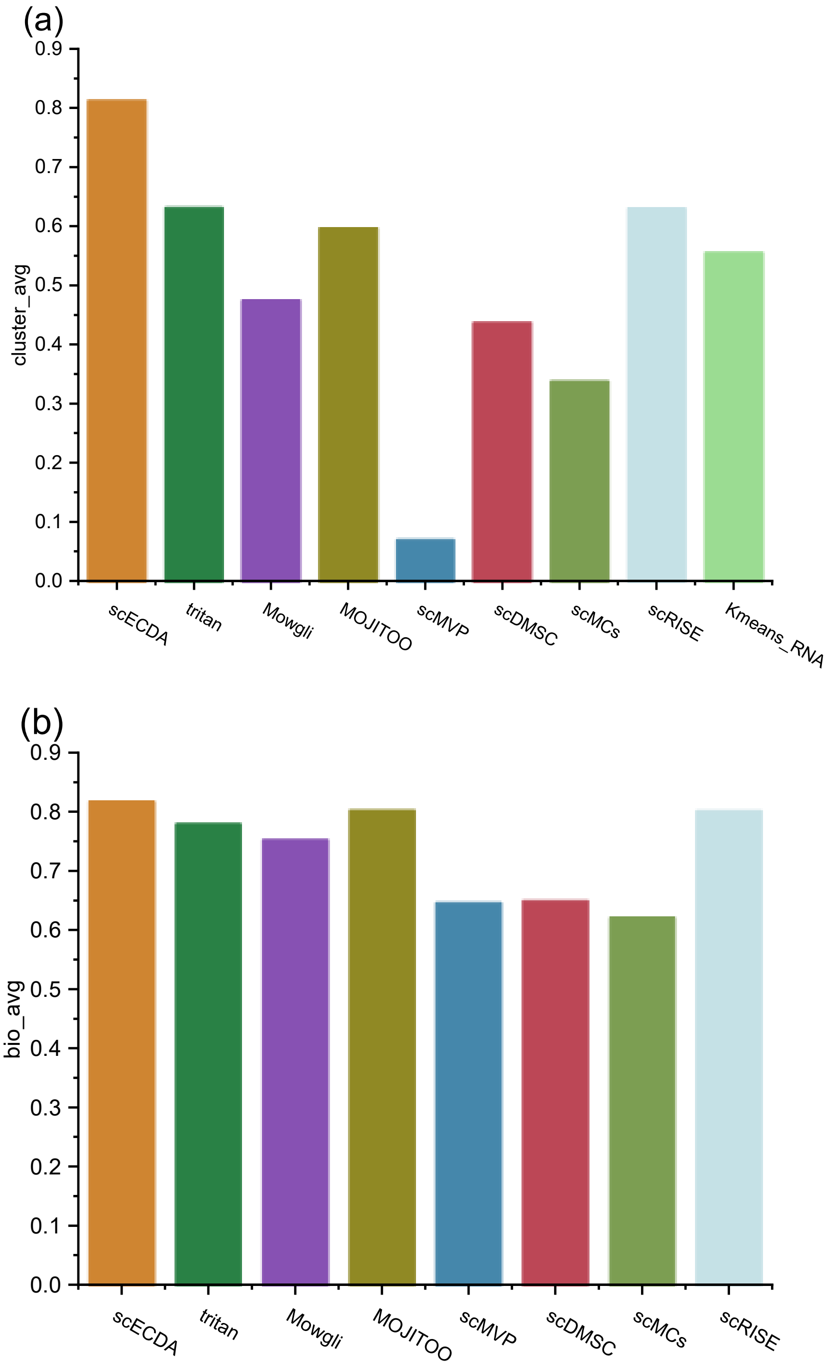

Fig S7. Evaluate the clustering accuracy and the ability to preserve biological specificity of different methods on the CITE_BMNC dataset.

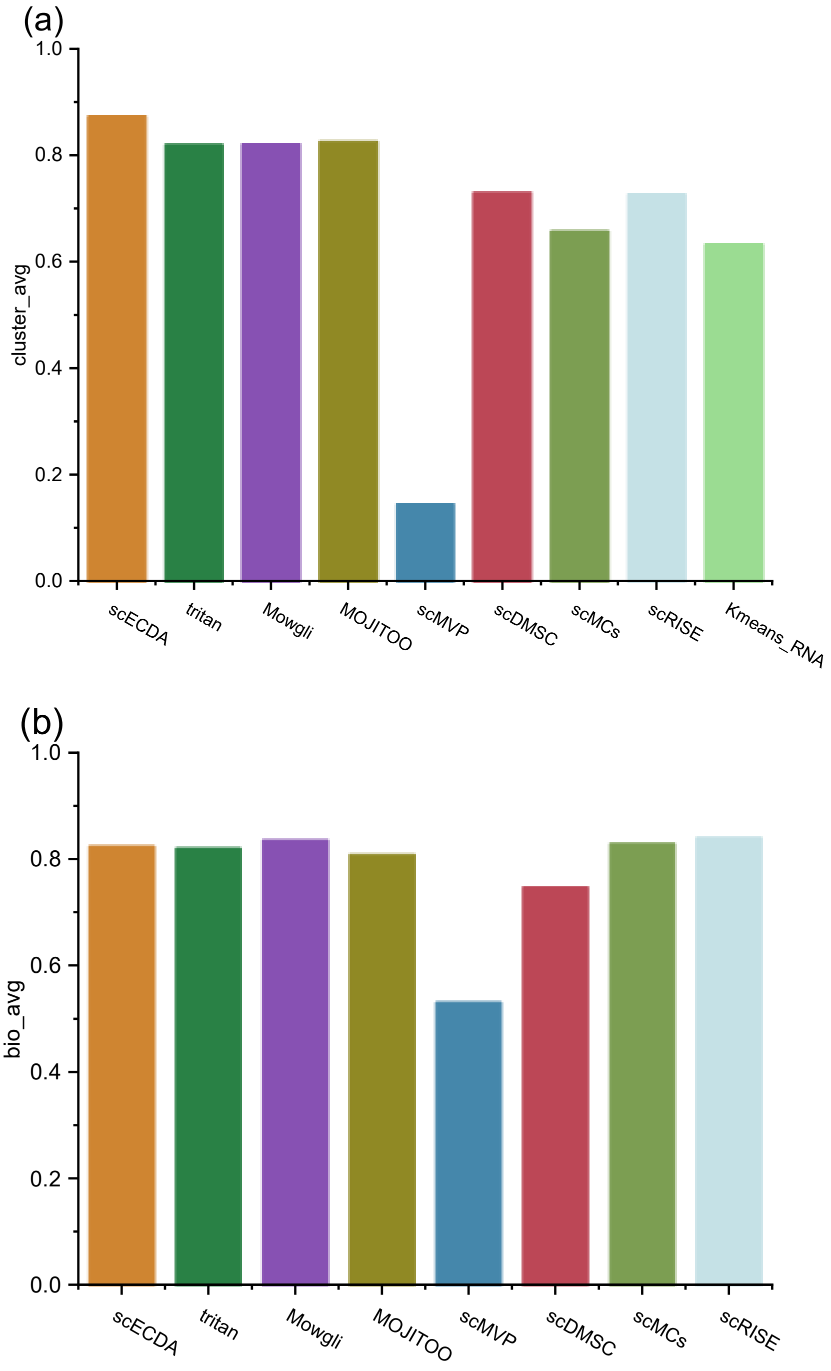

Fig S8. Evaluate the clustering accuracy and the ability to preserve biological specificity of different methods on the CITE _PBMC10x dataset.

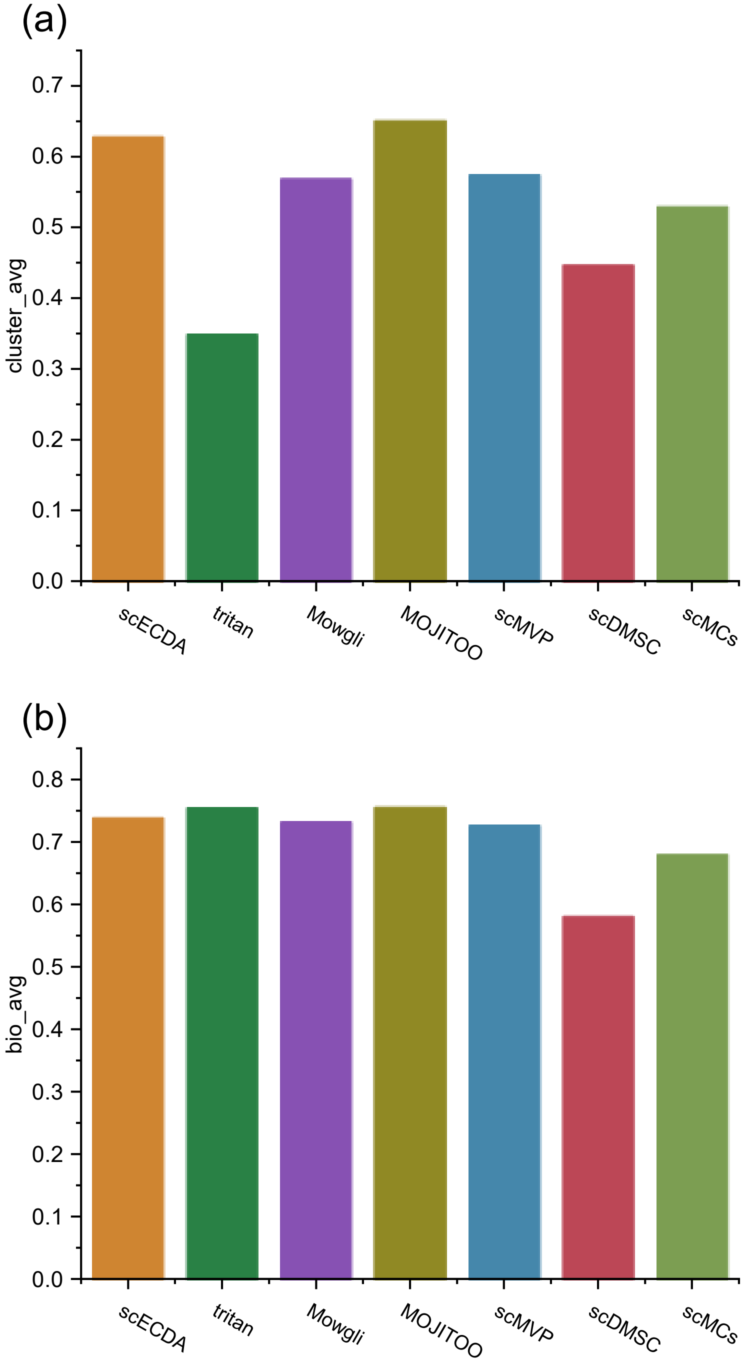

Fig S9. Evaluate the clustering accuracy and the ability to preserve biological specificity of different methods on the Tea_PBMC(RNA+ADT) dataset.

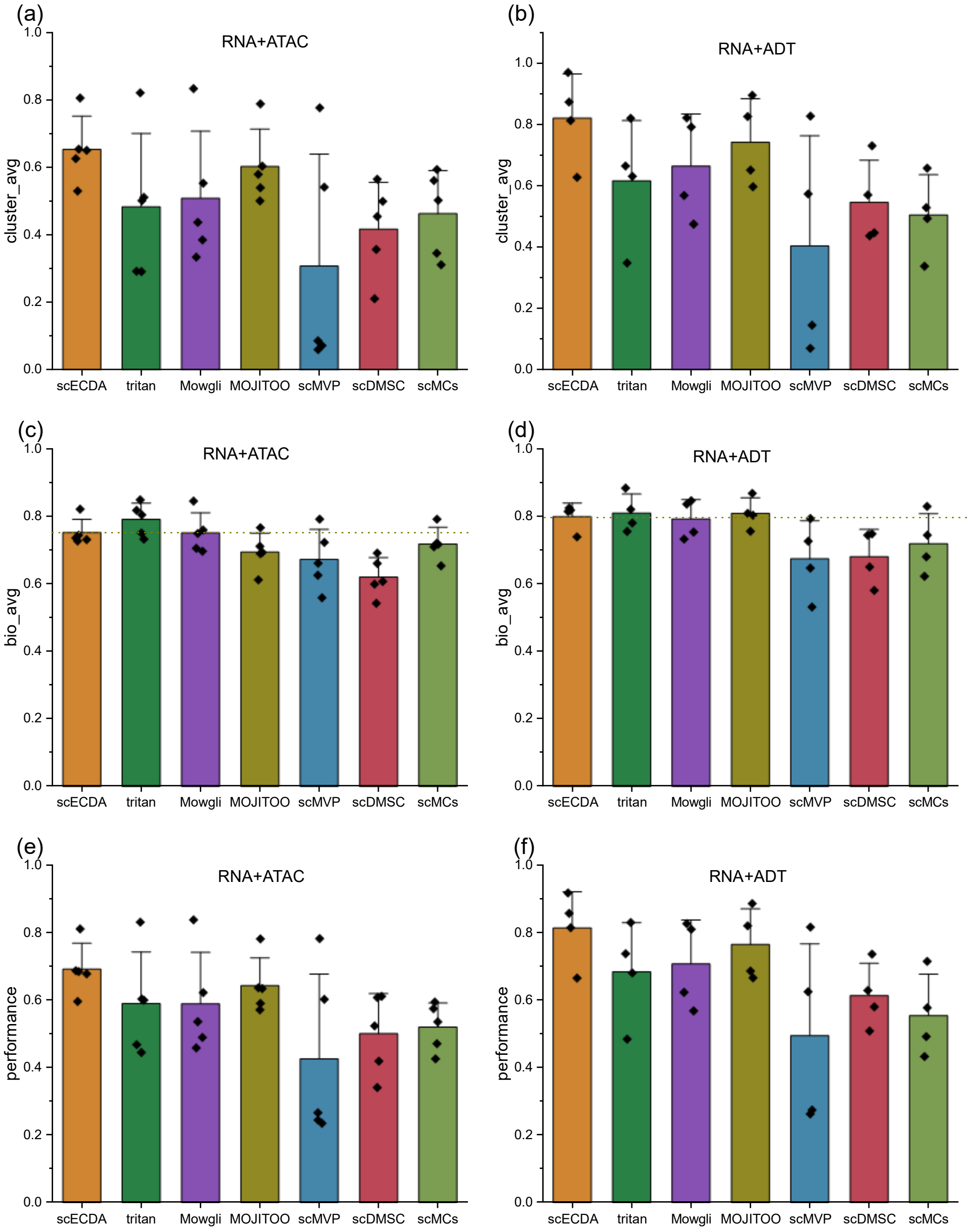

Fig S10. Evaluate the clustering accuracy, the ability to preserve biological specificity, and the overall performance of different methods on the (RNA+ADT) datasets, (RNA+ATAC) datasets and 8 all datasets.

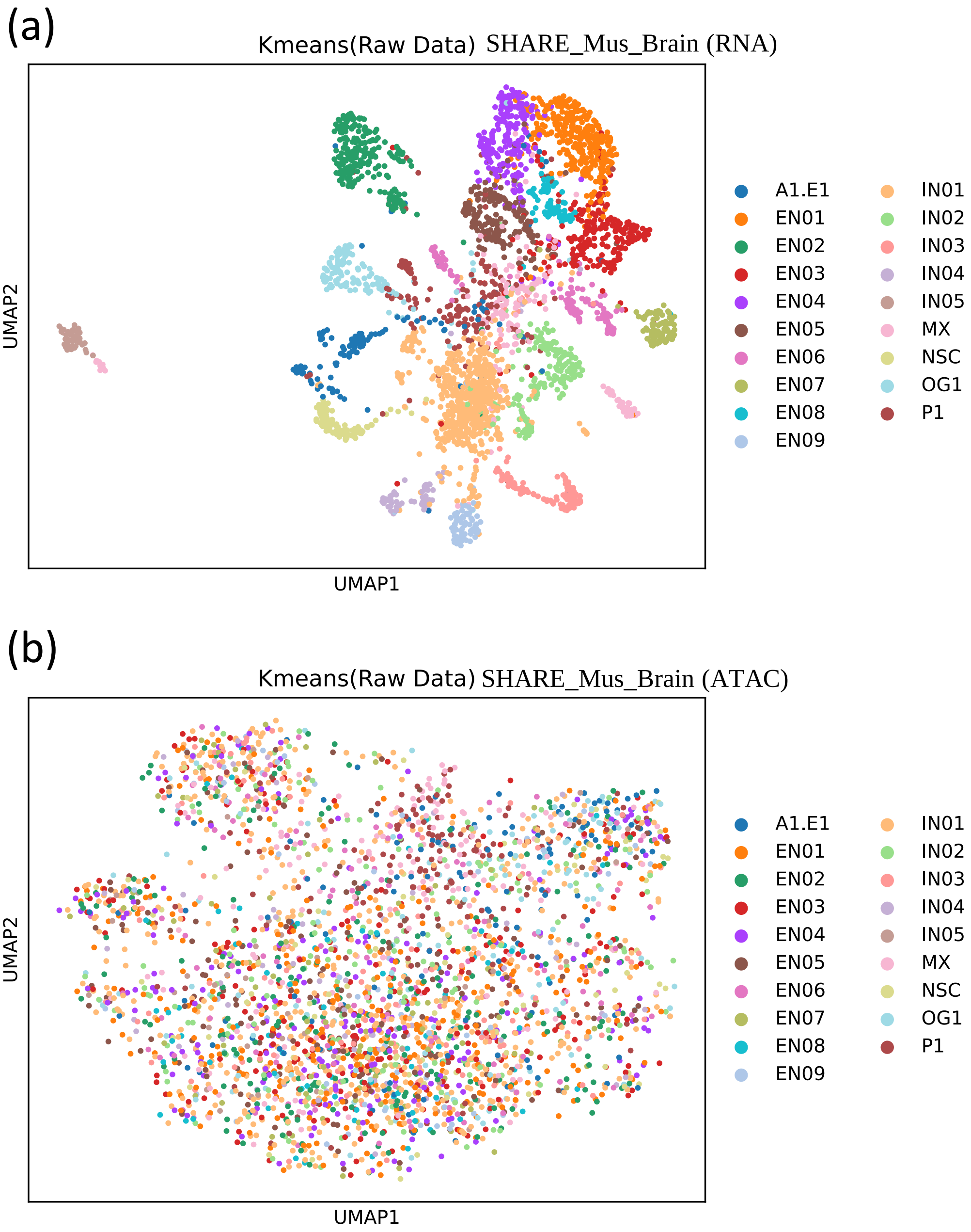

Fig S11. Umap Plot of SHARE_Mus_Brain.

(a)

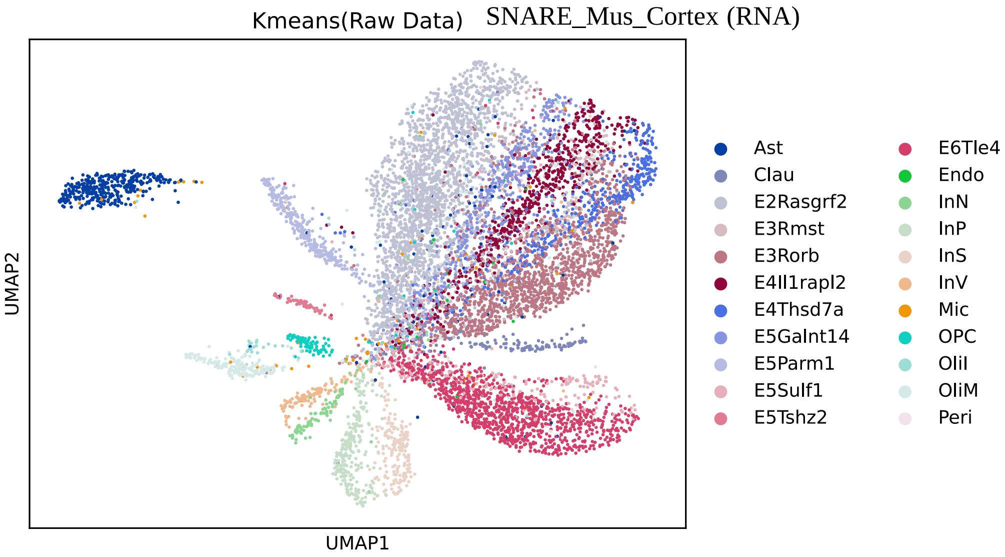

(b)

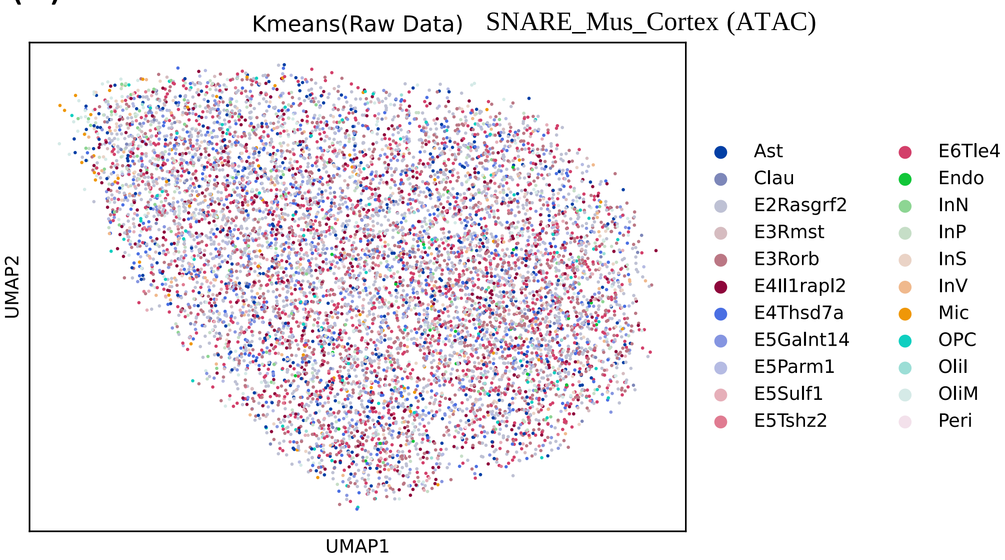

Fig S12. Umap Plot of SNARE_Mus_Cortex.

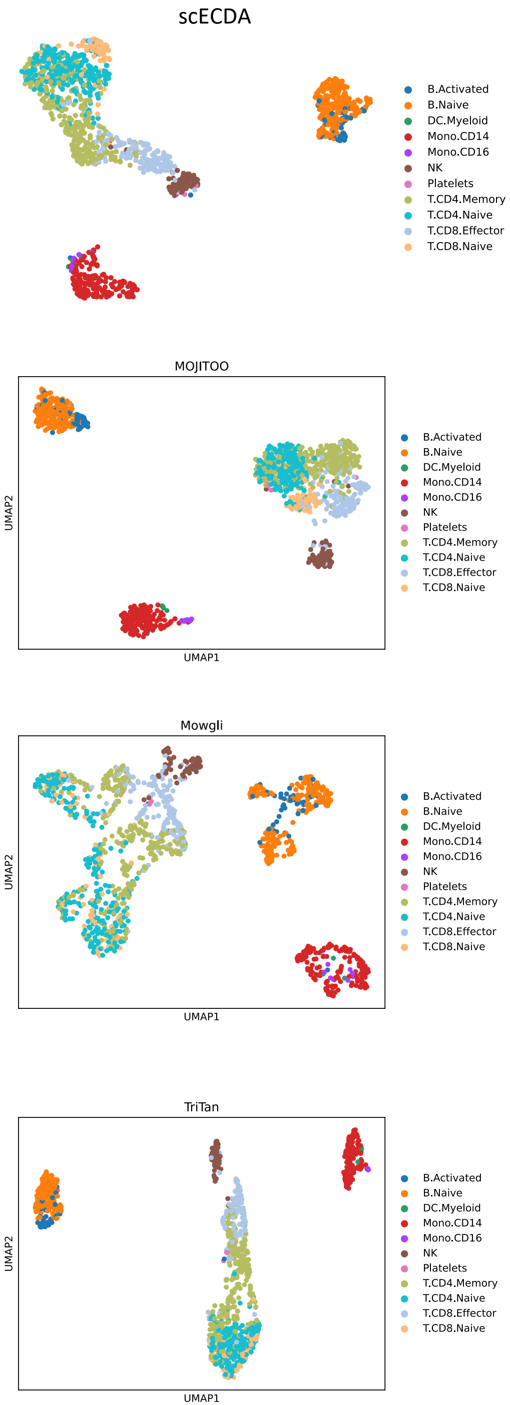

Fig S13. Tea_PBMC Latent feature distribution of scEDCA, MOJITOO, Mowgli, TriTan respectively.

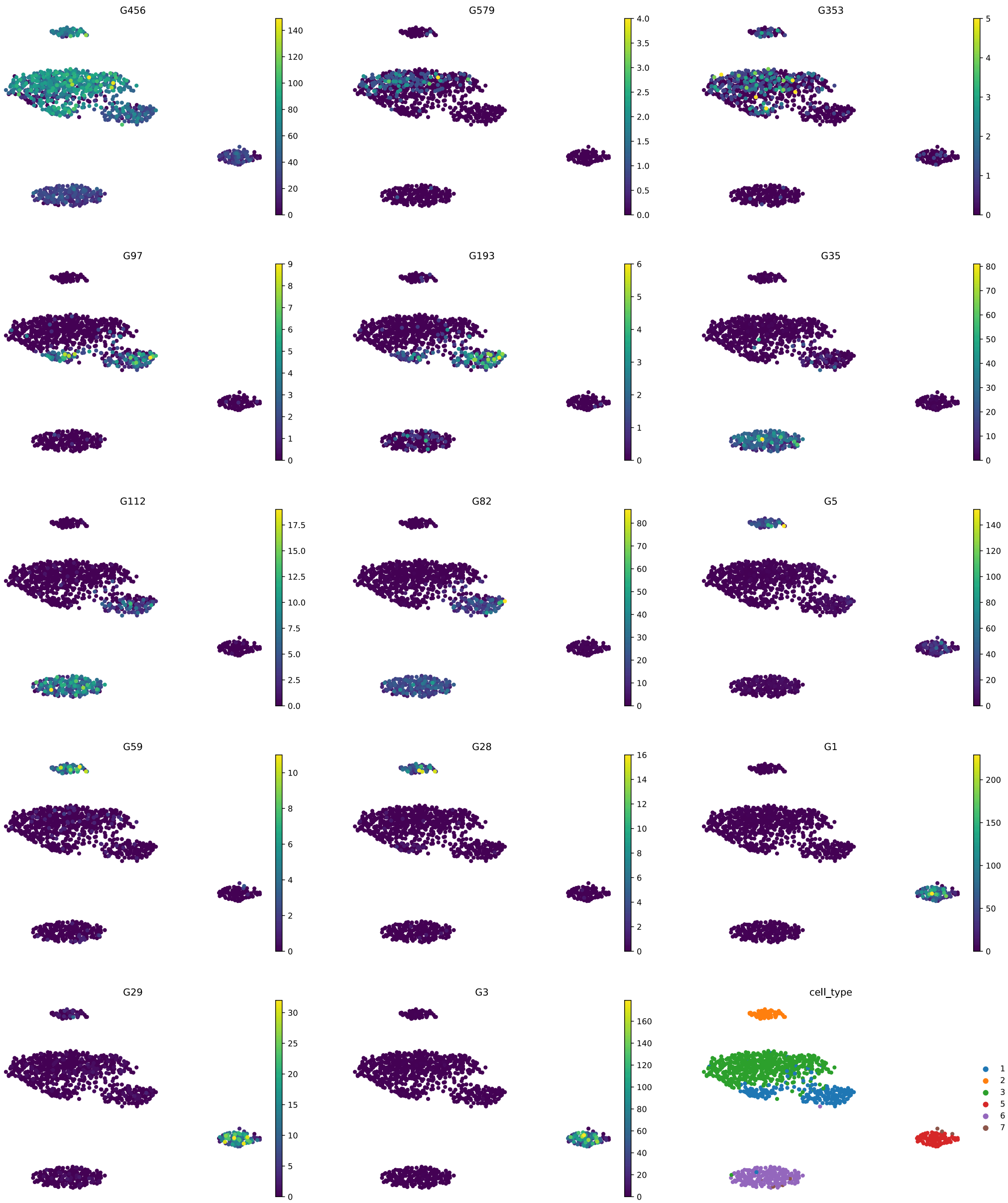

Fig S14. Distribution of selected differential proteins for cell types in CITE _PBMC_Inhouse dataset.

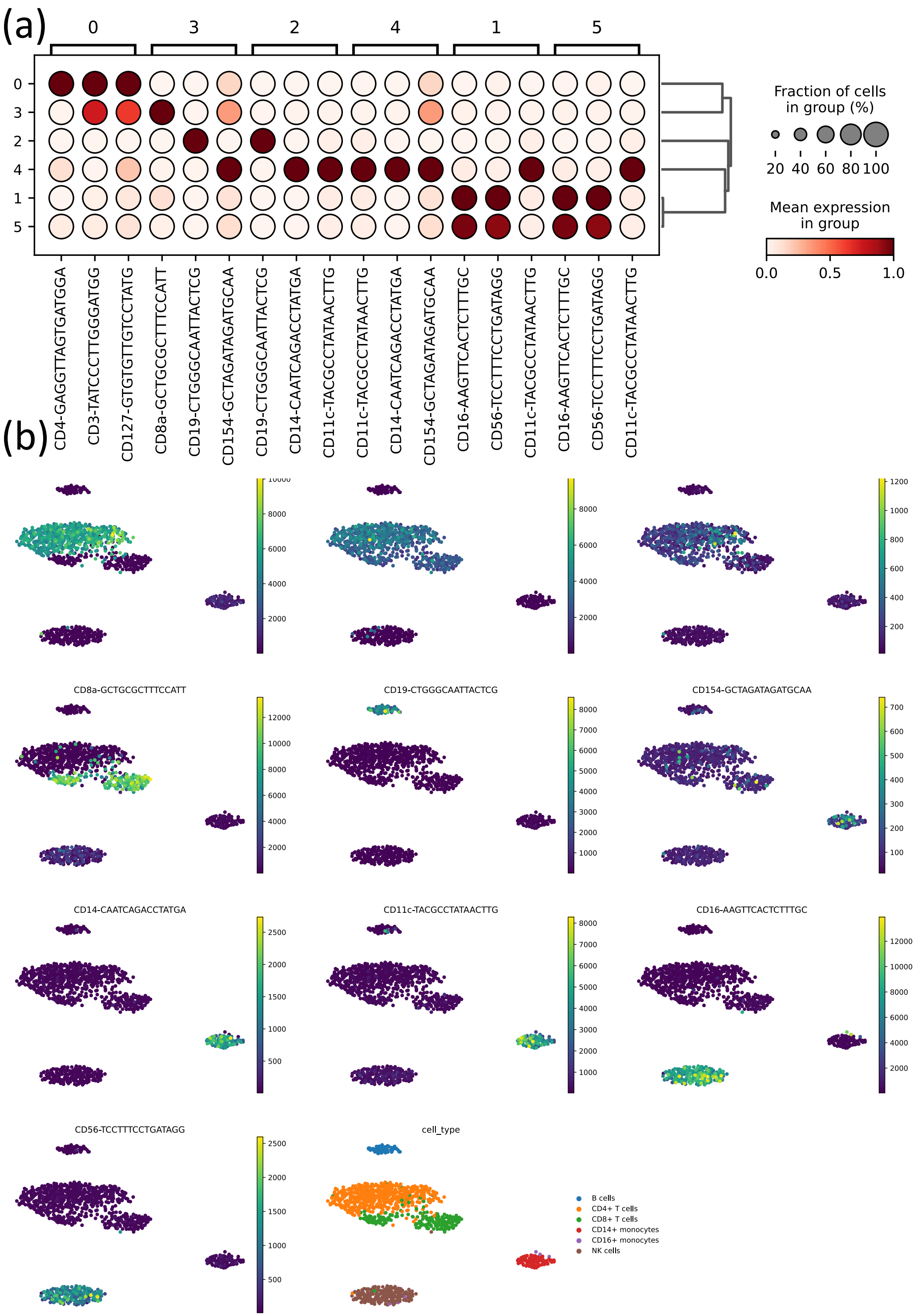

Fig S15. (a)dot plots of levels and (b)distribution of selected differential proteins for cell types in Inhouse dataset.

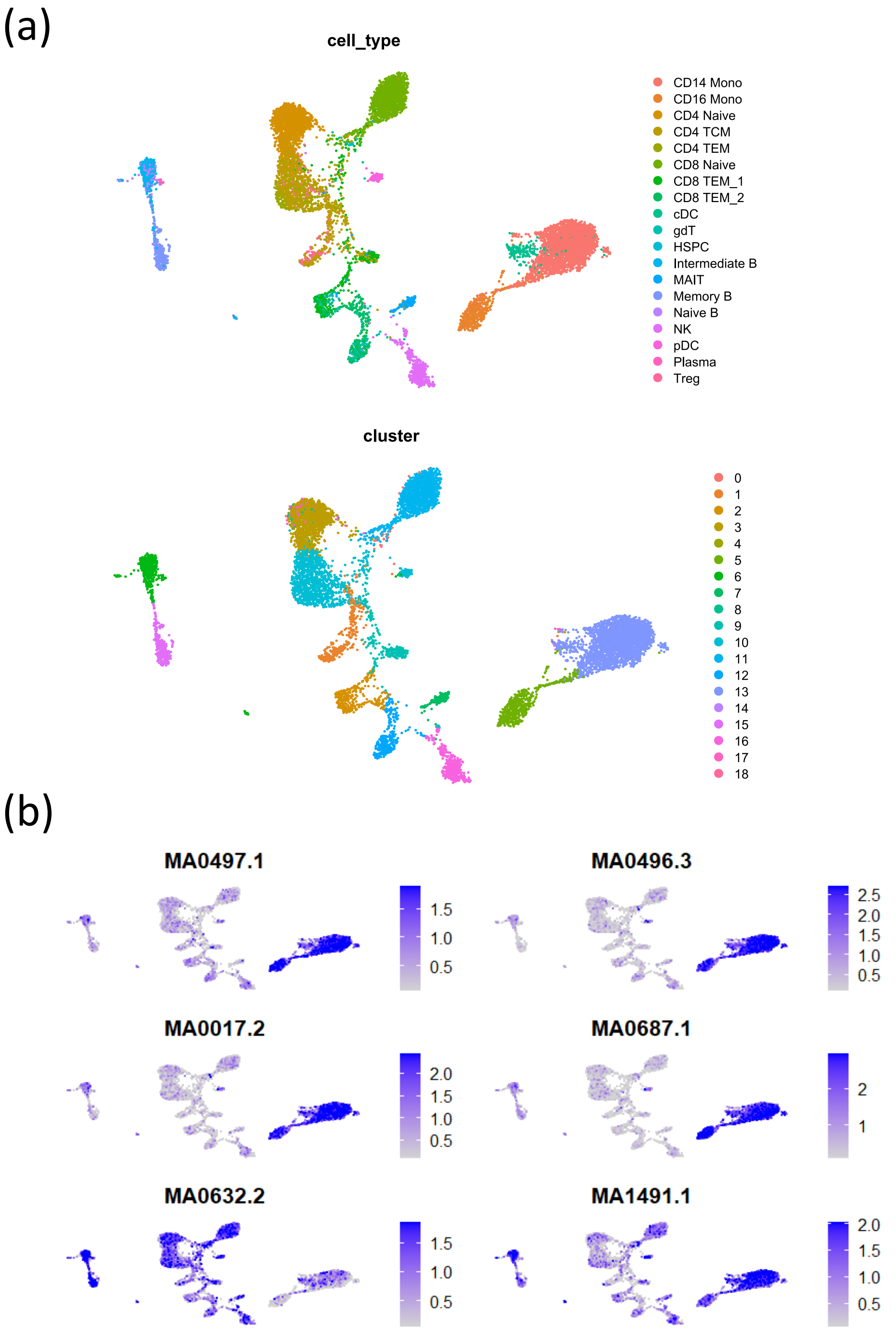

Fig S16. (a)UMAP plots of cell type and predicted cluster distribution. (b)distribution of selected enriched motifs in 10x Multiome_PBMC10x dataset.

MA0497.1

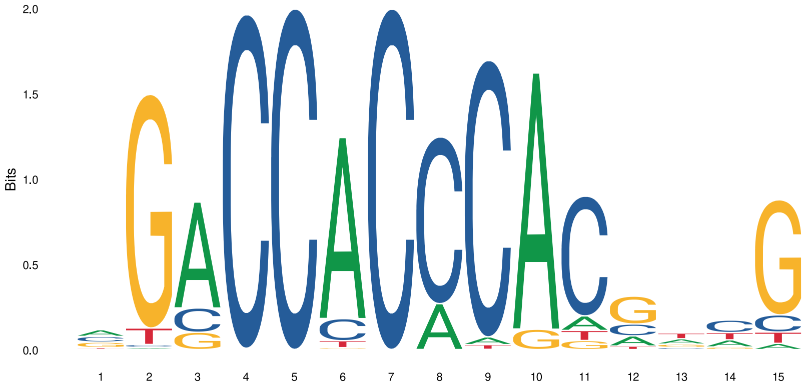

MA0497.1

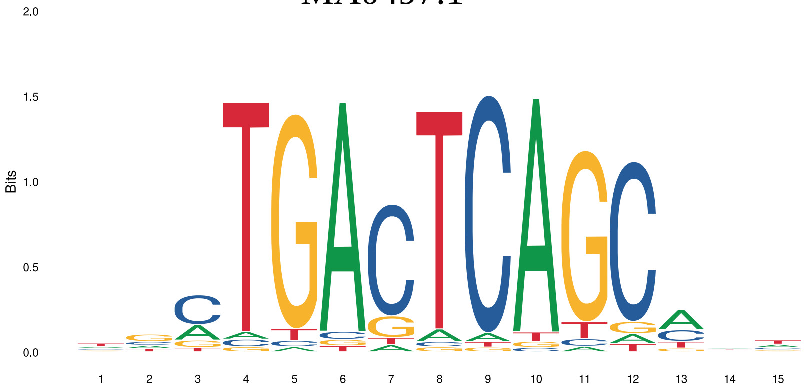

MA0632.2

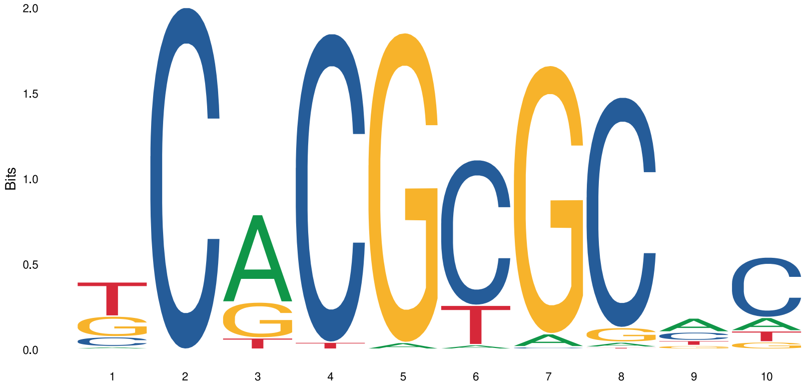

Fig S17. Sequence of transcription factor MA0497.1, MA0497.1, MA0632.2 binding motif

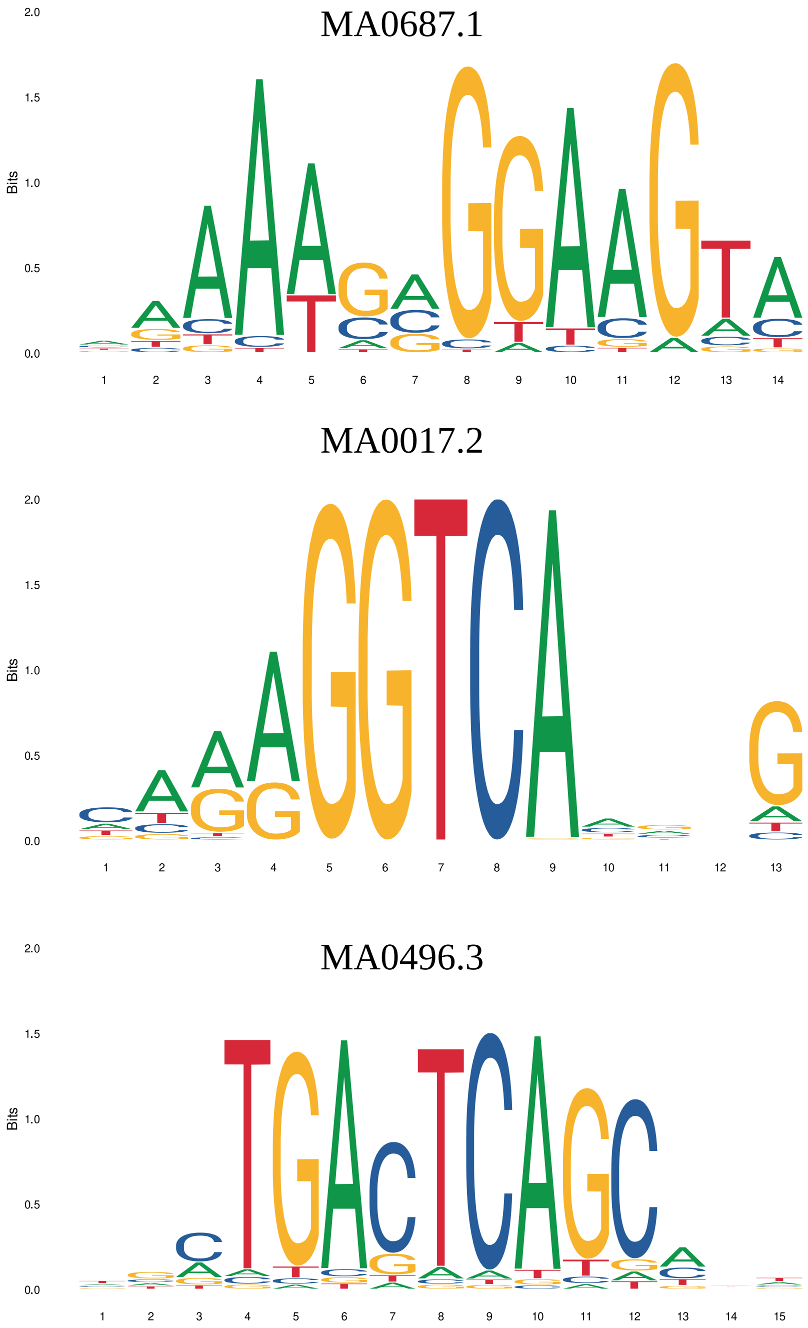

Fig S18. Sequence of transcription factor MA0687.1, MA0017.2, MA0496.3 binding motif

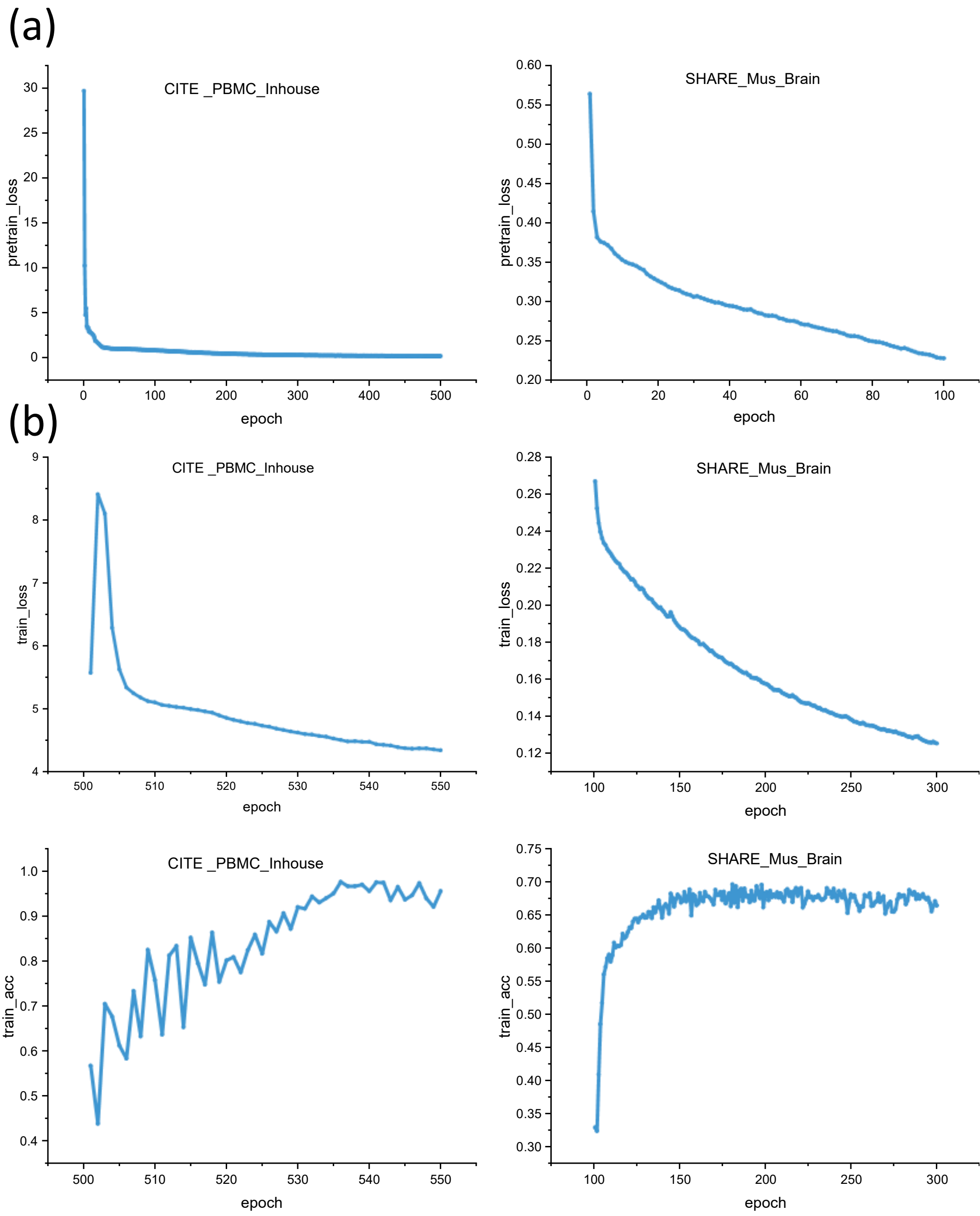

Fig S19. (a) Pretraining loss curve of CITE_PBMC_Inhouse and SHARE_Mus_Brain. (b) Training loss curve and accuracy curve of CITE_PBMC_Inhouse and SHARE_Mus_Brain.

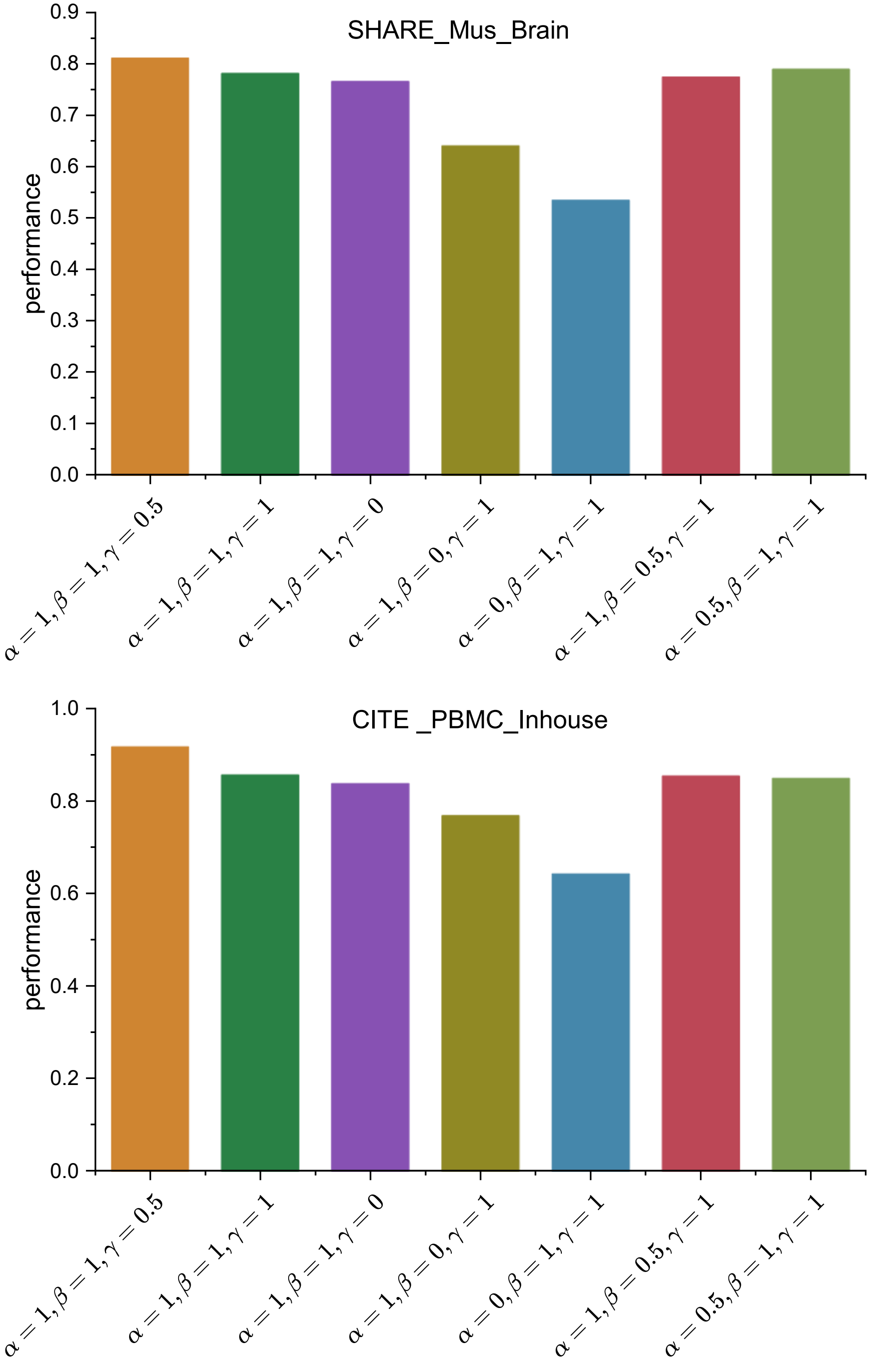

Fig S20. The trade-off parameters α, β, γ of three loss functions.

Supplementary Table 1. The summary of datasets

Dataset	Samples	Lables	RNA.dim	ATAC.dim	ADT.dim	RNA zero ratio	ATAC zero ratio
SNARE_Mus_Cortex(Chen, Lake and Zhang, 2019)	9190	22	28930	56167	\	0.9675	0.9725
CITE _PBMC_Inhouse(Wang, et al., 2020)	1182	6	33538	\	10	0.9063	\
CITE_BMNC(Lin, et al., 2022)	30672	27	17009	\	25	0.9021	\
10x Multiome_PBMC10x (Jiang, et al., 2023)	9631	19	29095	107194	\	0.9341	0.9310
CITE _PBMC10x(Cao and Gao, 2022)	6661	7	33538	\	14	0.8698	\
SHARE_Mus_Brain (Moroney, et al., 2020)	3293	19	21127	428041	\	0.9222	0.9975
Tea_PBMC (Liu, Huang and Yang, 2023)	1262	11	9855	14732	46	0.8911	0.7818
SHARE_Mus_skin_filtered(Ma, et al., 2020)	4619	5	3000	15000	\	0.9497	0.9869
10x Multiome _BMMC(Luecken, et al., 2021)	69249	22	13431	116490	\	0.9172	0.9692

Dataset	Species	Organization	Protocol
SNARE_Mus_Cortex(Chen, Lake and Zhang, 2019)	Mus musculus	Cortex	SNARE-seq
CITE _PBMC_Inhouse(Wang, et al., 2020)	Homo Sapiens	Peripheral Blood Mononuclear Cell	CITE-seq
CITE_BMNC(Lin, et al., 2022)	Homo Sapiens	Bone Marrow Mononuclear Cell	CITE-seq
10x Multiome_PBMC10x (Jiang, et al., 2023)	Homo Sapiens	Peripheral Blood Mononuclear Cell	CITE-seq
CITE _PBMC10x(Cao and Gao, 2022)	Homo Sapiens	Peripheral Blood Mononuclear Cell	10x Multiome
SHARE_Mus_Brain (Moroney, et al., 2020)	Mus musculus	Brain	SNARE-seq
Tea_PBMC (Liu, Huang and Yang, 2023)	Homo Sapiens	Peripheral Blood Mononuclear Cell	Tea-seq
SHARE_Mus_skin_filtered(Ma, et al., 2020)	Mus musculus	Skin	SHARE-seq
10x Multiome _BMMC(Luecken, et al., 2021)	Homo Sapiens	Bone Marrow Mononuclear Cells	10x Multiome

Supplementary Table 2. Validates the effectiveness of the differential attention mechanism-based feature fusion module, data augmentation module, and contrastive learning module on the Mouse Brain and Inhouse datasets.

Datasets	Inhouse				Mouse Brain			
Metrics	ARI↑	NMI↑	ACC↑	PUR↑	ARI↑	NMI↑	ACC↑	PUR↑
①diff	0.949	0.9193	0.9579	0.9748	0.5056	0.618	0.6729	0.7024
②self	0.6649	0.6812	0.7589	0.89	0.3771	0.5593	0.5408	0.6289
③con+diff	0.9556	0.9229	0.967	0.9814	0.525	0.6438	0.6887	0.7127
④con+self	0.7755	0.8147	0.8528	0.9323	0.4015	0.5766	0.5677	0.6483
⑤aug+self	0.7904	0.8346	0.8621	0.9357	0.4178	0.5817	0.5715	0.6486
⑥all	**0.9674**	**0.9407**	**0.9755**	**0.9941**	**0.5418**	**0.6577**	**0.6957**	**0.7227**

The table entries are defined as follows: ①diff: Removes the contrastive learning module while retaining the differential attention mechanism-based fusion module; ②self: Removes the contrastive learning module and

replaces the differential attention mechanism-based fusion module with a self-attention mechanism-based fusion module; ③con+diff: Removes the data augmentation module while retaining the contrastive learning module and the differential attention mechanism-based fusion module; ④con+self: Removes the data augmentation module, retains the contrastive learning module, and replaces the differential attention mechanism-based fusion module with a self-attention mechanism-based fusion module; ⑤aug+self: Replaces the differential attention mechanism-based fusion module with a self-attention mechanism-based fusion module while retaining the data augmentation and contrastive learning modules; ⑥all: Retains all modules.

Supplementary Table 3. scECDA hyperparameter settings

Dataset	RNA.latent.dim	ATAC.laten.dim	ADT.latent.dim	Pretrain_epoch	Train_epoch	lr	noise
SNARE_Mus_Cortex	500	500	\	200	50	0.0003	0.003
CITE _PBMC_Inhouse	500	\	500	500	50	0.0003	0.003
CITE_BMNC	500	300	\	30	250	0.0003	0.002
10x Multiome_PBMC10x	400	\	50	30	100	0.0003	0.003
CITE _PBMC10x	500	500	\	200	30	0.0003	0.002
SHARE_Mus_Brain	512	128	\	100	200	0.0003	0.002
Tea_PBMC	512	512	256	20	60	0.0003	0.003
SHARE_Mus_skin_filtered	512	512	\	90	200	0.0003	0.003
10x Multiome _BMMC	500	500	\	110	50	0.0003	0.0002

The principles, parameter Settings and data preprocessing of eight comparison methods

scMVP automatically learns a common latent representation for scRNA-seq and scATAC-seq data through a clustering consistency-constrained multi-view variational autoencoder (VAE) model. It imputes each single-layer dataset from the common latent embedding of the multi-omics data via layer-specific data generation processes, including a transformer's self-attention-based scATAC generation channel and a mask attention-based scRNA generation channel. scMVP is implemented using the PyTorch framework. The model is trained with the Adam optimizer, a batch size of 128, a learning rate of 5.0e−3, and a maximum of 30 iterations. All datasets are trained for 30 epochs. scMVP requires the raw gene expression matrix for RNA and the TF-IDF transformed ATAC expression matrix as input. The default dimensionality for data integration is 20, and we followed the original workflow for processing. To achieve optimal results, scMVP requires pre-specifying the number of clusters, which we set equal to the true number of cell types.

Mowgli is a novel integrative matrix factorization (MF) method for single-cell multi-omics data, combining integrative Non-negative Matrix Factorization (integrative NMF) with Optimal Transport (OT). Mowgli is implemented using the PyTorch framework. The default dimensionality for data integration is 15, and data preprocessing is performed using the Scanpy and Muon toolkits. The default number of training epochs is 25.

TriTan is an efficient joint factorization method for single-cell multi-omics data. Its three-matrix factorization approach aids in clustering cells, identifying signature features for each cell type, and uncovering feature associations across omics. TriTan is implemented using the PyTorch framework. For scRNA-seq matrices, TriTan performs sc.pp.filter_genes(), sc.pp.normalize_total(), sc.pp.log1p(), and sc.pp.highly_variable_genes() to filter

low-quality genes. For the scATAC-seq modality, TriTan first applies TF-IDF transformation, followed by sc.pp.log1p() and sc.pp.highly_variable_genes() to filter low-quality peaks. The first phase of training runs for 20 epochs, and the second phase runs for 30 epochs. The default dimensionality for data integration is 2.

MOJITOO is an efficient method based on Canonical Correlation Analysis (CCA) to learn a shared latent space for any single-cell multimodal data protocol. The canonical components can be interpreted as factors and used to characterize feature relevance by relating features across modalities. Additionally, it provides an approach to estimate the size of the latent space after a single execution of CCA. MOJITOO is implemented in R and also offers a Python version; we used the Python implementation. MOJITOO reduces the dimensionality of RNA data using PCA and ATAC data using LSI, both defaulting to 50 dimensions. No processing is performed for ADT data. The dimensionality for data integration and the number of training epochs are determined by the MOJITOO algorithm itself.

scDMSC, an unsupervised clustering algorithm based on deep multi-view subspace learning. This approach coordinates the heterogeneity of omics data through weighted reconstruction and employs deep subspace learning to identify shared latent features, elucidating the correlations among the omics. scDMSC is implemented based on python, and RNA data is processed by the Scanpy package. The ATAC data is first transformed by TF-IDF and then processed by LSI for dimensionality reduction. During the pre-training phase, the Adam optimizer is adopted with a learning rate of 0.001 for 100 epochs. Mean squared error loss is employed to obtain the initial weights. In the training process, the model uses the Adadelta optimizer with an initial learning rate of 0.01 and a decay factor of 0.95 for clustering. Additionally, Cosine Annealing is applied to improve convergence speed and stability [40]. The spectral clustering parameter $r0$ is set to 8.0. Since the number of training rounds was not mentioned, we follow the default value of 60 in the code, and the default dimension for data integration is 32.

scRISE, a deep clustering method for scRNA-seq data. The model consists of two main modules: an iterative smoothing module based on graph autoencoders designed to denoise the data and refine the pairwise similarity in turn to gradually incorporate cell structural features and enrich the data information; and a self-supervised discriminative embedding module with adaptive similarity threshold for partitioning samples into correct clusters. scRISE was implemented in Python 3 (version 3.8) using PyTorch (version 2.0). We initially set the learning rate as lr=0.001, epoch=100, and batch size = 256, and then used Adam optimizer. The size of the adaptive encoder was set as 32. The learning rate for the adaptive encoder was lr=0.0005, and an initial threshold was set $r_{pos}^{st} = 0.0015$ and $r_{neg}^{st} = 0.3$, while the final threshold is set to $r_{pos}^{ed} = 0.001$ and $r_{neg}^{ed} = 0.7$, the number of update iterations (T) to 40, and the batch size for sample pairs to 10,000. We trained the model for 400 epochs using the Adam optimizer. The hyperparameters α and β were both set to 10. The default dimension for data integration is 32. Those hyperparameters and procedures are followed by original publication.

scMCs, a single-cell data fusion based multiple clustering approach. The main idea is to design an information extraction and fusion module to finely process the individuality and commonality learned from heterogeneous omics, and construct a more comprehensive and informative representation for single-cell multi-omics data fusion, clustering, and multiple clustering. scMCs is divided into five training stages, and the parameter configuration follows the original paper.

K-means, a widely applicable unsupervised clustering algorithm suitable for various scenarios. RNA Data Preprocessing: The RNA raw expression matrix is first normalized using the scanpy.pp.normalize_total function. The normalized expression matrix is then log-transformed (natural logarithm) using scanpy.pp.log1p. Highly variable genes (HVGs) are selected based on the Seurat algorithm (flavor='seurat') via the scanpy.pp.highly_variable_genes function. Principal Component Analysis (PCA) is performed on the expression

matrix of HVGs using scanpy.pp.pca, extracting the top n_comps=60 principal components. ATAC Data Preprocessing: The preprocessing steps for ATAC data are identical to those for RNA data. ADT Data Preprocessing: Only log-transformation (scanpy.pp.log1p) is applied to the ADT data.

The Leiden algorithm is employed for clustering the integrated features, implemented via the scanpy.tl.leiden function. To ensure a fair comparison, the resolution parameter is adjusted such that the number of cluster labels matches the number of true labels.

Analyze the biomarkers identified by scECDA on the CITE _PBMC_Inhouse dataset

The biological biomarkers identified by scECDA in the InHouse dataset are illustrated in Figure 6. For instance, the genes S100A9, DUSP1, and LYZ are highly expressed exclusively in CD14+ monocytes. CD14+ monocytes can differentiate into macrophages or dendritic cells, a process dependent on the sequential activation of the MAPK pathway (e.g., ERK driving cell proliferation and differentiation). DUSP1(Teng, et al., 2018) dephosphorylates ERK in a "pulse-like" manner, potentially suppressing unnecessary signal extension during early differentiation while allowing specific gene expression (e.g., pro-inflammatory factors) in later stages, thereby balancing differentiation efficiency with functional specificity. S100A9(Ryckman, et al., 2003; Simard, Girard and Tessier, 2010; Vogl, et al., 2004), a member of the S100 calcium-binding protein family, is highly expressed in monocytes and is closely associated with innate immune functions. During infection, CD14+ monocytes rapidly release S100A9, which induces neutrophil chemotaxis through CXC chemokine receptor 4 (CXCR4) or the RAGE receptor, forming an inflammatory cascade. S100A9 activates the SYK/PI3K/AKT pathway, enhancing neutrophil phagocytic efficiency and lysosome enzyme release, thereby directly participating in pathogen clearance. S100A9 itself can induce neutrophil degranulation via a MAPK/ERK-dependent mechanism (forming a feedback loop with DUSP1's MAPK regulation). LYZ(Safran, et al., 2010) encodes lysozyme and is a key biomarker in the monocyte-macrophage system, with its high expression directly reflecting the phagocytic function of monocytes. Lysozyme hydrolyzes bacterial cell wall peptidoglycans (e.g., N-acetylglucosamine bonds) to kill Gram-positive bacteria and activates the complement cascade. When released extracellularly (e.g., lactoferrin), lysozyme enhances anti-tumor activity (e.g., via the TLR4/NF-κB pathway) and interacts with adaptive immunity mediated by monocytes. The genes GNLY, GZMB, and CST7 are highly expressed in NK cells, with GZMB and CST7 also showing high expression in some CD8+ T cells. GNLY(Sun, et al., 2021) encodes a core bactericidal protein in NK cell cytoplasmic granules that directly damages cellular membranes to injure intracellular pathogens (e.g., Mycobacterium tuberculosis). As the "first line of defense" in innate immunity, NK cells must rapidly respond to pathogen infections, and high expression of GNLY ensures efficient elimination of infected cells, preventing pathogen dissemination. GZMB triggers apoptosis by cleaving GSDME and is a core mechanism for NK cells to eliminate virus-infected cells and tumor cells(Hameed, et al., 1988; Krähenbühl, et al., 1988; Poe, et al., 1991). In cytotoxic phases (e.g., in the liver cancer microenvironment), CD8+ T cells eliminate tumor cells through GZMB-mediated apoptosis, with expression levels positively correlated with cytotoxic phenotypes (e.g., co-expression of GZMH and GNLY). CST7(Sawyer, et al., 2021), a member of the cystatin family, inhibits the activity of cysteine proteases (e.g., granule proteases) to prevent excessive inflammation and self-tissue

damage. In NK cell cytotoxicity, CST7 may balance the activities of GZMB and GNLY, avoiding cell self-destruction due to lysosomal membrane rupture. GNLY and GZMB (granule-derived) are both present in NK cell cytotoxic granules, with GNLY damaging target cell membranes and GZMB activating GSDME to induce apoptosis, forming a coordinated "membrane perforation + content release" killing mechanism. This synergy is particularly important in NK cell responses to viral or intracellular bacterial infections. In the effector phase of CD8+ T cells, CST7 regulates the switch between apoptosis and necrosis by inhibiting GSDME cleavage, maintaining the precision of immune responses. The genes CD74, CD79A, and CD79B are specific to B cells. CD79A and CD79B, as core components of the B-cell receptor (BCR) signaling complex, drive B-cell responses to antigens and their differentiation(Hashimoto, Chiorazzi and Gregersen, 1994; Müller, Cooper and Terhorst, 1992; Wood Jr, et al., 1993). CD74 stabilizes heterodimers of MHC class II molecules and directs their transport to lysosomes/endosomes, ensuring efficient antigen processing and presentation. As professional antigen-presenting cells (APCs), B cells must efficiently process extracellular antigens (e.g., viral proteins) to activate T cells(Downs-Canner, et al., 2022), with CD74 playing a key role in this process. CD74 also strengthens B-cell functions in antigen presentation and antiviral immunity by regulating antigen presentation pathways, reinforcing the central role of B cells in adaptive immunity(Bruchez, et al., 2020). This mechanism provides potential therapeutic targets for B-cell-related diseases (e.g., lymphoma, autoimmune diseases), such as ADC drugs targeting CD79B.

Explanation of Q3+1.5*IQR：

In statistics, the Interquartile Range (IQR) is used to define outliers with a coefficient of 1.5 (i.e., the boundaries are set at $Q1 - 1.5 \times IQR$ and $Q3 + 1.5 \times IQR$. This choice is based on a comprehensive consideration of the sensitivity of normal distributions to outliers and practical robustness. The $1.5 \times IQR$ rule was proposed by John Tukey during the development of the box plot (Box Plot). This value stems from observations of real-world data distributions, particularly in small samples or non-normally distributed data, where the IQR is less sensitive to extreme values (since $Q1$ and $Q3$ are based on quantiles), and the 1.5 multiplier accommodates most distribution types.

Assuming the data follows a normal distribution, substituting the coefficient 1.5 into the boundary formulas yields:

Lower bound: $Q1 - 1.5 \times IQR \approx (\mu - 0.675\sigma) - 1.5 \times 1.35\sigma = \mu - 2.7\sigma$,

Upper bound: $Q3 + 1.5 \times IQR \approx (\mu + 0.675\sigma) + 1.5 \times 1.35\sigma = \mu + 2.7\sigma$.

Under a normal distribution, the probability of data falling within $\mu \pm 2.7\sigma$ is approximately 99.3% (i.e., only about 0.7% of data lies outside this range). This boundary aligns closely with the 3σ principle of normal distributions (99.73% coverage), effectively identifying extreme values.

When calculating distances between cells after dimensionality reduction, only cells with distances exceeding $Q3 + 1.5 \times IQR$ are filtered out, accounting for approximately 0.35% of the data. For example, in a dataset of 10,000 cells, only about 35 cells would be filtered. For other distributions, the number of filtered cells remains around 35. Since outlier cells exhibit significantly larger distances compared to normal cells, the probability of mistakenly filtering normal cells is negligible.

These outlier cells—often identified as low-quality in other workflows—are filtered only during the training process.

During testing, all cells in the dataset, including these outliers, are retained for clustering. Filtering such anomalous cells enhances the stability of model training and accelerates convergence.

REFERENCE

Bruchez, A., *et al.* MHC class II transactivator CIITA induces cell resistance to Ebola virus and SARS-like coronaviruses. *Science* 2020;370(6513):241-247.

Cao, Z.-J. and Gao, G. Multi-omics single-cell data integration and regulatory inference with graph-linked embedding. *Nature Biotechnology* 2022;40(10):1458-1466.

Chen, S., Lake, B.B. and Zhang, K. High-throughput sequencing of the transcriptome and chromatin accessibility in the same cell. *Nature biotechnology* 2019;37(12):1452-1457.

Downs-Canner, S.M., Meier, J., Vincent, B.G. and Serody, J.S. B cell function in the tumor microenvironment. *Annual review of immunology* 2022;40(1):169-193.

Hameed, A., Lowrey, D.M., Lichtenheld, M. and Podack, E.R. Characterization of three serine esterases isolated from human IL-2 activated killer cells. *Journal of Immunology (Baltimore, Md.: 1950)* 1988;141(9):3142-3147.

Hashimoto, S., Chiorazzi, N. and Gregersen, P.K. The complete sequence of the human CD 79b (lgβ/B29) gene: identification of a conserved exon/intron organization, immunoglobulin-like regulatory regions, and allelic polymorphism. *Immunogenetics* 1994;40:145-149.

Jiang, H., Zhan, S., Ching, W.-K. and Chen, L. Robust joint clustering of multi-omics single-cell data via multi-modal high-order neighborhood Laplacian matrix optimization. *Bioinformatics* 2023;39(7):btad414.

Krähenbühl, O., *et al.* Characterization of granzymes A and B isolated from granules of cloned human cytotoxic T lymphocytes. *Journal of immunology (Baltimore, Md.: 1950)* 1988;141(10):3471-3477.

Lin, X., Tian, T., Wei, Z. and Hakonarson, H. Clustering of single-cell multi-omics data with a multimodal deep learning method. *Nature communications* 2022;13(1):7705.

Liu, C., Huang, H. and Yang, P. Multi-task learning from multimodal single-cell omics with Matilda. *Nucleic Acids Res* 2023;51(8):e45-e45.

Luecken, M.D., *et al.* A sandbox for prediction and integration of DNA, RNA, and proteins in single cells. In, *Thirty-fifth conference on neural information processing systems datasets and benchmarks track (Round 2)*. 2021.

Ma, S., *et al.* Chromatin potential identified by shared single-cell profiling of RNA and chromatin. *Cell* 2020;183(4):1103-1116. e1120.

Moroney, J.B., *et al.* Integrative transcriptome and chromatin landscape analysis reveals distinct epigenetic regulations in human memory B cells. *Nature communications* 2020;11(1):5435.

Müller, B., Cooper, L. and Terhorst, C. Cloning and sequencing of the cDNA encoding the human homologue of the murine immunoglobulin‐associated protein B29. *European journal of immunology* 1992;22(6):1621-1625.

Poe, M., *et al.* Human cytotoxic lymphocyte granzyme B. Its purification from granules and the characterization of substrate and inhibitor specificity. *Journal of Biological Chemistry* 1991;266(1):98-103.

Ryckman, C., *et al.* Proinflammatory activities of S100: proteins S100A8, S100A9, and S100A8/A9 induce neutrophil chemotaxis and adhesion. *The Journal of Immunology* 2003;170(6):3233-3242.

Safran, M., *et al.* GeneCards Version 3: the human gene integrator. *Database* 2010;2010:baq020.

Sawyer, A.J., Garand, M., Chaussabel, D. and Feng, C.G. Transcriptomic profiling identifies neutrophil-specific upregulation of cystatin F as a marker of acute inflammation in humans. *Frontiers in immunology* 2021;12:634119.

Simard, J.-C., Girard, D. and Tessier, P.A. Induction of neutrophil degranulation by S100A9 via a MAPK-dependent mechanism. *Journal of leukocyte biology* 2010;87(5):905-914.

Sun, Y., *et al.* Single-cell landscape of the ecosystem in early-relapse hepatocellular carcinoma. *Cell* 2021;184(2):404-421. e416.

Teng, F., *et al.* DUSP1 induces apatinib resistance by activating the MAPK pathway in gastric cancer. *Oncology reports* 2018;40(3):1203-1222.

Vogl, T., *et al.* MRP8 and MRP14 control microtubule reorganization during transendothelial migration of phagocytes. *blood* 2004;104(13):4260-4268.
Wang, X., *et al.* BREM-SC: a bayesian random effects mixture model for joint clustering single cell multi-omics data. *Nucleic Acids Res* 2020;48(11):5814-5824.
Wood Jr, W.J., *et al.* Isolation and chromosomal mapping of the human immunoglobulin-associated B29 gene (IGB). *Genomics* 1993;16(1):187-192.
